# Associations between ankle strength and physical performance in healthy individuals: a systematic review

**DOI:** 10.3389/fphys.2026.1863201

**Published:** 2026-07-15

**Authors:** Tim S. Babu, Premraj Saini, Rohit K. Thapa, João Bruno, Liam Houlton, Paul J. Byrne, Exal Garcia-Carrillo

**Affiliations:** 1 Symbiosis School of Sports Sciences, Symbiosis International (Deemed University), Pune, India; 2 CIPER, Faculdade de Motricidade Humana, Universidade de Lisboa, Cruz-Quebrada-Dafundo, Portugal; 3 Faculty of Sport Sciences and Physical Education, CIPER, University of Coimbra, Coimbra, Portugal; 4 Portuguese Air Force, Avenida da Força Aérea Portuguesa, Amadora, Portugal; 5 School of Sport and Health Sciences, Cardiff Metropolitan University, Cardiff, United Kingdom; 6 Faculty of Sport, Health and Fitness, City College Plymouth, Plymouth, United Kingdom; 7 Department of Health and Sport Sciences, Faculty of Health Science, South East Technological University (Kilkenny Road Campus), Carlow, Ireland; 8 Department of Physical Activity Sciences, Faculty of Education Sciences, Universidad Católica del Maule, Talca, Chile; 9 Department of Physical Activity Sciences, Universidad de Los Lagos, Osorno, Chile

**Keywords:** athletic performance, exercise, human physical conditioning, motor activity, movement, muscle strength

## Abstract

**Aim:**

This systematic review aimed to assess the association between ankle muscle (i.e., plantarflexor, dorsiflexor, invertor and evertor muscles) strength and measures of physical performance in healthy individuals.

**Methods:**

A systematic search was conducted across three electronic databases (PubMed, Scopus, and Web of Science) up to 30th April 2025. Studies investigating the relationship between ankle strength and physical performance outcomes were included. Study eligibility was determined using the PECOS framework and methodological quality was assessed using a modified Downs and Black checklist.

**Results:**

Thirty-four studies with a total of 2,275 participants met the eligibility criteria. 11 studies examined the relationship between ankle strength and static balance, 5 studies focused on dynamic balance, 8 on jump performance, 6 on sprint performance, and 4 on reactive strength index (RSI). Additionally, individual studies explored associations with deceleration, reaction time, muscular endurance, and change of direction speed. Statistically significant associations indicated that ankle plantarflexor and dorsiflexor strength showed positive associations with jump height (*r* = 0.32-0.85), sprint speed (*r* = 0.36 - 0.86), and RSI (*r* = 0.35-0.526), and significant negative correlations with static (*r* = -0.28 to -0.925) and dynamic balance measures (*r* = -0.24 to -0.91). The strength of associations ranged from small to very strong (*r* = 0.32–0.95).

**Conclusion:**

Ankle muscle strength is significantly associated with a wide range of physical performance outcomes in healthy individuals. These findings suggest that ankle muscle strength may be an important characteristic to consider in physical training programs.

## Introduction

The ankle is a complex joint structure consisting of the talocrural, subtalar, and inferior tibiofibular joints (Medina Mckeon and Hoch, 2019) and serves as a crucial kinetic link between the lower leg and foot, allowing the lower limb to interact with the ground (Brockett and Chapman, 2016). The surrounding ankle muscles, including plantarflexors, dorsiflexors, invertors and evertors are major contributors to functional and athletic performance (Brockett and Chapman, 2016). Adequate ankle muscle strength has been linked to effective locomotion (Tavakkoli Oskouei et al., 2021), maintaining balance (Bok et al., 2013), and fall prevention (Cattagni et al., 2014). Regarding athletic performance, it contributes to running biomechanics (Kerrigan et al., 2009), agility (Laplaca and Seedman, 2021), stability (De Villiers and Venter, 2014), jump performance (Ding et al., 2024), change of direction speed (CODS) (Kozinc et al., 2021), and strength and power expression (Goldmann et al., 2013).

Ankle strength can be assessed through various methods (e.g., isometric and dynamic), each focusing on different neuromuscular and biomechanical characteristics. Isometric tests are commonly used to measure ankle strength in previous literature due to their simplicity and reliability (Jiménez-Ávila et al., 2025). In contrast, dynamic assessments may provide greater ecological validity due to the closer resemblance to athletic movements (Wilson and Murphy, 1996). Moreover, individual ankle muscle groups perform distinct biomechanical roles that may differentially influence performance outcomes (Neptune et al., 1999; Monteleone et al., 2014; Moisan et al., 2022). These functional differences underscore the importance of muscle-specific ankle assessment. Additionally, physical performance is a multifactorial construct commonly measured using sprinting, jumping, reactive strength, CODS, balance, and strength performance, all of which require different neuromuscular and biomechanical characteristics (Pleša et al., 2022). Accordingly, these outcomes are assessed using a wide range of field- and laboratory-based tests (e.g., sprint tests, agility protocols, and countermovement jumps [CMJ], and single-leg stance balance tests, one-repetition maximum [1RM] testing) (Pleša et al., 2022).

Consequently, increasing research interest has focused on the association between ankle muscle strength and physical performance outcomes. In university-level athletes, moderate to strong positive associations have been found between plantarflexion (PF) and dorsiflexion (DF) strength and single-leg jump tasks (Singh et al., 2022), likely indicating enhanced ground force production via the coordinated net muscle moments of the hip, knee, ankle and toe flexors (Yamauchi and Ishii, 2007; Yamauchi and Koyama, 2020). Similarly, inter-limb asymmetry in PF strength has been linked to impaired CMJ performance among adolescent basketball players, highlighting the impact ankle strength asymmetry on jump performance and the role of the PF in power transmission (Ding et al., 2024). In athletes (i.e., youth soccer and basketball players, elite sprinters, and male floorball players), greater isokinetic and isometric PF strength have been linked to superior sprint and CODS performance (İnal et al., 2012; Kozinc et al., 2021; Trajković et al., 2021; Chen et al., 2025; Vecbērza et al., 2025). For example, studies have shown that higher PF torque and reactive strength enhance acceleration, cutting, and jumping ability (İnal et al., 2012; Trajković et al., 2021; Vecbērza et al., 2025), while stronger PF in both limbs contributes to more effective horizontal deceleration during short sprints (Chen et al., 2025). Moreover, ankle strength has also been associated with balance performance, with PF and DF strength being significantly related to static and reactive balance metrics (Abd El-Kader, 2014). In relation to ankle invertors, Soares et al. (2024) reported a significant association between invertor peak torque and dynamic balance outcomes. The results indicated that stronger ankle musculature improves the control of torque and intrinsic ankle stiffness, reducing quiet standing sway and responses to perturbations in dynamic tasks in an improved manner (Sakanaka et al., 2021).

However, despite the considerable number of studies in the association between ankle strength and performance (Wyrick, 1969; Lin et al., 2009; İnal et al., 2012; Abd El-Kader, 2014; Cattagni et al., 2014; Cobb et al., 2014; Kim and Park, 2015; Hagen et al., 2020; Yoshizawa et al., 2020; Feehan et al., 2022; Yoshizawa and Yoshida, 2022; Hébert-Losier et al., 2023; Ding et al., 2024; Kadlubowski et al., 2024; Kayhan et al., 2024; Chen et al., 2025; Vecbērza et al., 2025), the literature remains difficult to interpret due to large methodological heterogeneity, as studies varies in the ankle muscle groups, strength assessment methods (i.e., isometric, isokinetic, or isotonic), participant’s characteristics and physical performance outcomes which may lead to broad range of reported finding and complicates the interpretation.

Although a previous review found moderate association between PF strength and balance (*r* = 0.34; *rz* = 0.35; 95% confidence interval [CI]=006-0.63; *p* = 0.02) and a weaker association with walking (Tavakkoli Oskouei et al., 2021) in healthy individuals. Nonetheless, to our knowledge, no systematic review has comprehensively synthesized evidence across multiple physical performance domains (i.e., sprint, jump, RSI, CODS, balance) in relation to ankle strength in healthy individuals. Moreover, most of the previous studies have relied on an isometric strength testing method to measure ankle strength, which may not fully represent how ankle strength contributes to dynamic activity (Wilson and Murphy, 1996). Consequently, it remains uncertain whether individual ankle muscle groups exhibit consistent association with specific physical performance outcomes or not.

Therefore, this review aims to provide guidance for practitioners by synthesizing evidence that can inform both practical decision-making and future scientific investigations. Coaches, athletic trainers, and clinicians may rely on this evidence to inform training strategies, improve performance assessment, and refine injury prevention approaches. At the same time, researchers can use these findings to standardize methodologies in future studies to address unresolved questions in the field. Thus, the current systematic review aims to aggregate the literature on the association of ankle muscle strength (e.g., PF, DF, inversion [IV] and eversion [EV]) with measures of physical performance in healthy individuals.

## Methods

### Protocol and registration

This systematic review followed the updated 2020 Preferred Reporting Items for Systematic Reviews and Meta-Analyses (PRISMA) in Exercise, Rehabilitation, Sport medicine and Sports Science (PERSiST) guidelines (Page et al., 2021; Ardern et al., 2022). The study protocol was prospectively registered in the Open Science Framework platform (OSF), DOI https://doi.org/10.17605/OSF.IO/729YJ (Project link: https://osf.io/j5t2g).

### Eligibility criteria

The eligibility criteria used for this systematic review were based on the participants, exposure, comparator, outcome, and study design (PECOS) approach (**Table 1**). There were no restrictions on age, sex, or physical fitness level for the study’s inclusion. However, only studies involving healthy and physically active individuals or athletes [Tier 1 or above based on the Participant Classification Framework (Mckay et al., 2022)] were considered eligible. Additionally, studies were included if they investigated the association between concentric, eccentric, or isometric strength of the ankle muscles, specifically the dorsiflexors, plantar flexors, invertors, or evertors and various measures of physical performance (both health- and skill-related fitness) such as (but not limited to) aerobic endurance, body composition, CODS, linear sprints, maximal strength (e.g., 1RM squat), or jumps (e.g., CMJ, squat jumps [SJ], drop jump [DJ], RSI). All outcomes reported by the studies were extracted and presented. No restrictions were applied to the language of publication during the selection process.

**Table 1:** Selection criteria used in this systematic review.

Category	Inclusion criteria	Exclusion criteria
Population	Healthy individuals.	Clinical populations, specially-abled but healthy human populations (e.g., para-athletes), individuals with musculoskeletal disorders, or neurological conditions.
Exposure	Studies that assessed the association of concentric, eccentric, or isometric strength with dorsiflexors, plantar flexors, invertors, and evertors in relation to measures of physical performance.	Studies that did not assess the association of ankle strength measures with measures of physical performance.
Comparator	Not applicable.	Not applicable.
Outcome	Studies that reported health- (e.g., maximal strength) and/or performance-related (e.g., static balance) fitness outcomes.	Studies that did not report health or performance-related outcomes.
Study design	Cross-sectional studies.	Intervention studies.

### Information sources

The literature search was carried out in three major electronic databases, i.e., PubMed, Scopus, and Web of Science. The studies published from the establishment of each database up to April 30, 2025, were considered for inclusion in this systematic review.

### Search strategy

The keywords collected were based off research papers published previously and related to ankle strength and controlled vocabulary (e.g., Medical Subject Headings: MeSH). The final keywords and search string were prepared through consensus between the authors. The search strategy for specific databases is provided in **Table 2**.

**Table 2:** Search strategy used at different databases.

Date of search	30th April 2025	30th April 2025	30th April 2025
Databases	PubMed	WoS (Core Collection)	Scopus
Keywords	“ankle strength”, “plantar flexors strength”, “dorsiflexors strength”, “invertors strength”, “evertors strength”, “isometric ankle strength”, “isokinetic ankle strength”, “physical performance”		
Database fields for the search	Title, abstract	Topic	Title, abstract, keywords

### Selection process

The study selection process was based on a two-stage approach, and two authors (TSB and PS) screened the identified studies for inclusion in the systematic review based on predefined eligibility criteria. In the first stage, duplicate records were removed, and the titles and abstracts of the remaining studies were screened to assess their relevance. In the second stage, the full text of the potentially eligible studies were reviewed for inclusion. Any disagreements between the two authors during the selection process were resolved through discussion with a third author (RKT) to reach a consensus. Inter-rater agreement between reviewers was examined using Cohen’s kappa coefficient for title/abstract screening and full-text review (κ = 0.77 and κ =0.92, respectively).

### Data collection process

Mean and standard deviations of the independent (i.e., ankle strength measures) and dependent (i.e., physical performance measures) variables were extracted from the included studies and were recorded in customized Microsoft Excel Sheets (Microsoft Corporation, Redmond, WA, USA). Similarly, correlation coefficients (e.g., Pearson’s *r*, Spearman’s ρ, or Kendall’s τ), along with corresponding *p*-values, sample sizes and confidence interval, were extracted. If required data were not clearly reported in the studies, corresponding authors were contacted to obtain the raw data. The lead author (TSB) was responsible for the initial data extraction, while another author (PS) independently verified its accuracy. Any disagreements were resolved through discussion and consensus with a third author (RKT).

### Data items

The data were extracted from retrieved studies for relevant health- and performance-related physical fitness outcome variables such as static and dynamic balance, linear sprint speed, vertical jump height (e.g., countermovement jump, squat jump), reaction time, deceleration, CODS, endurance, and maximal strength (e.g., 1RM squat). In addition, ankle strength assessment characteristics were extracted and classified based on the contraction mode (e.g, isometric, concentric, eccentric or isokinetic) and measurement approach (e.g., torque, force or repetition based measures), when reported. Article information (author name, journal name, year of publication), participants and tests (ankle strength and measures of physical performance), characteristics, equipment, manufacturer details, and statistical information were also recorded in the customized Microsoft Excel spreadsheets. Participants’ information included age, body mass, stature, sex, sport practiced, and participant classification (Mckay et al., 2022). Test information included the equipment used, including manufacturer details, criterion measure, test reliability, and value of measures (e.g., mean/SD or Median/IQR).

### Risk of bias assessments

To appraise each study’s methodological quality, a modified version of the Downs and Black Checklist (**Supplementary File S1**) was used (Downs and Black, 1998) in accordance with previous studies (Fox et al., 2018; Bujalance-Moreno et al., 2019; Jarvis et al., 2022). Two authors (TSB and PS) independently evaluated the risk of bias for each included study. Any differences in assessment were addressed via consensus and resolved with a third author (RKT) if necessary.

## Results

### Literature search results

**Figure 1:**
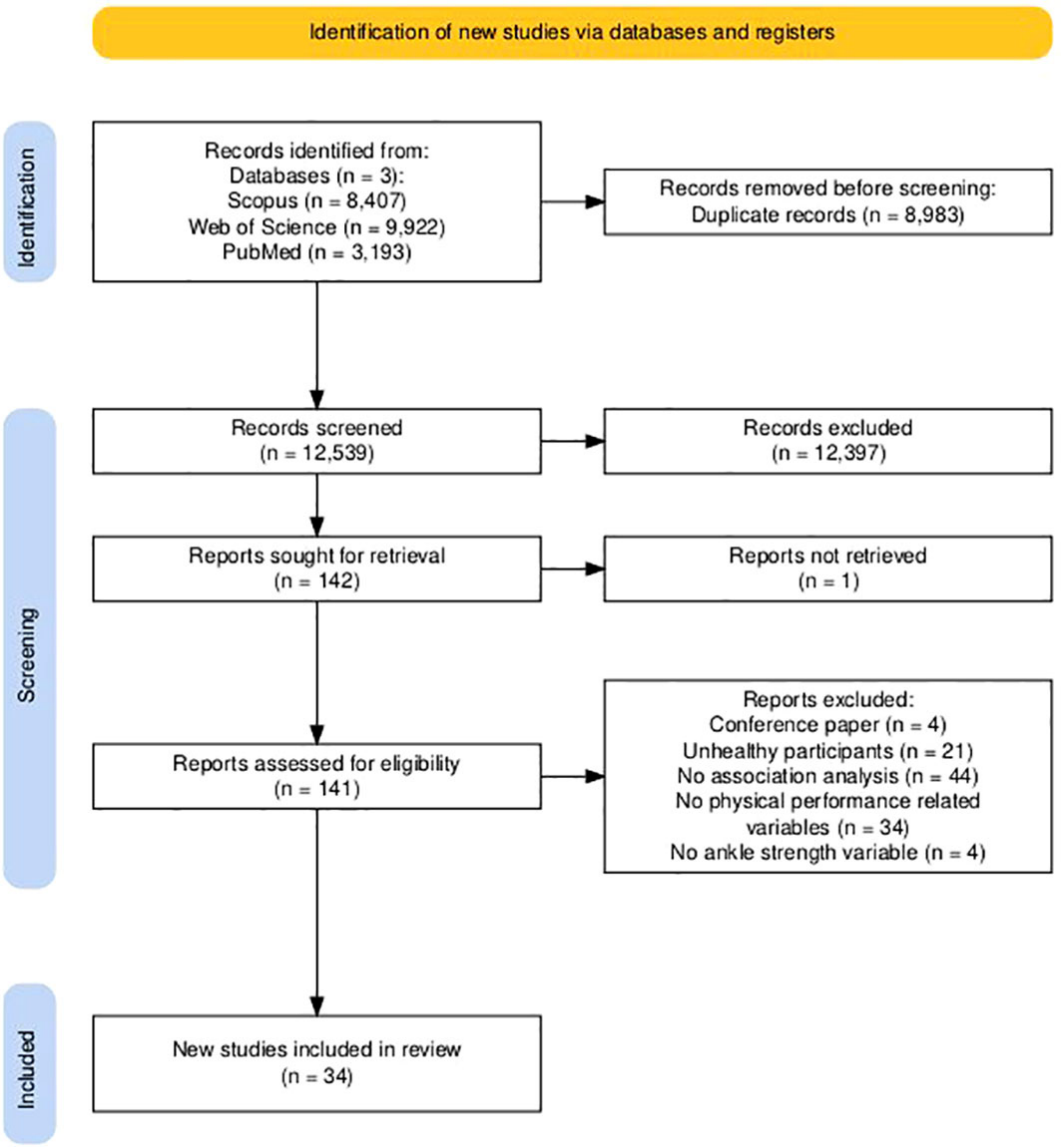
PRISMA flow diagram.

The initial search across different databases resulted in a total of 21,522 articles (**Figure 1**), of which 8,983 duplicates were removed. A further 12,397 articles were excluded based on abstract and title screening. Thereafter, a full text review was conducted on 142 articles and 34 studies were identified for inclusion. Participant characteristics and study characteristics are presented in **Tables 3**, **4**, respectively. **Table 4** includes the key dataset. Detailed information, including manufacturer details and non-significant associations, is provided in **Supplementary Files S2**, **S3**, respectively.

**Table 3:** Methodological quality assessment scores using the modified Downs and Black checklist.

Study	Reporting						External validity	Internal validity			Score
	1	2	3	4	5	6	7	8	9	10	
Abd El-Kader (2014)	+	+	+	+	+	–	?	+	+	+	8
Cattagni et al. (2014)	+	+	+	+	+	+	?	+	+	+	9
Chen et al. (2021)	+	+	+	+	+	+	?	+	+	+	9
Cobb et al. (2014)	+	+	+	+	+	–	?	+	+	+	8
Ding et al. (2024)	+	+	+	+	+	+	?	+	+	+	9
Feehan et al. (2022)	+	+	+	+	–	+	?	+	+	+	8
Hagen et al. (2020)	+	+	+	+	–	–	?	+	+	+	7
Hébert-Losier et al. (2023)	+	+	+	+	+	+	?	+	+	+	9
Hirono et al. (2020)	+	+	+	+	+	+	?	+	+	+	9
Lin et al. (2009)	+	+	+	+	+	–	?	+	+	+	8
İnal et al. (2012)	+	+	+	+	+	–	?	+	+	+	8
Kadlubowski et al. (2024)	+	+	+	+	+	–	?	+	+	+	8
Kayhan et al. (2024)	+	+	+	+	+	–	?	+	+	+	8
Kim and Park (2015)	+	+	–	+	+	–	+	+	+	+	8
Kouzaki and Shinohara (2010)	+	+	+	+	–	–	?	+	+	+	7
Kozinc et al. (2021)	+	+	+	+	+	+	+	+	+	+	10
Möck et al. (2018)	+	+	+	+	+	+	?	+	+	+	9
Möck et al. (2023)	+	+	+	+	+	+	?	+	+	+	9
Muehlbauer et al. (2013)	+	+	+	+	+	+	?	+	+	+	9
Muehlbauer et al. (2012)	+	+	+	+	+	+	?	+	+	+	9
Oshita and Yano (2012)	+	+	+	+	+	+	?	+	+	+	9
Ranisavljev et al. (2014)	+	+	+	+	+	+	?	+	+	+	9
Sara et al. (2021)	+	+	+	+	+	+	?	+	+	+	9
Shimizu et al. (2024)	+	+	+	+	+	–	?	+	+	+	8
Singh et al. (2022)	+	+	+	+	+	–	?	+	+	+	8
Słomka and Michalska (2024)	+	+	+	+	–	–	?	+	+	+	7
Genuario and Dolgener (1980)	+	+	–	+	+	–	–	–	+	+	6
Tao et al. (2020)	+	+	–	+	+	–	?	+	+	+	7
Trajković et al. (2021)	+	+	+	+	+	–	?	+	+	+	8
Vecbērza et al. (2025)	+	+	+	+	+	–	?	+	+	+	8
Wyrick (1969)	+	+	+	+	+	–	?	–	+	?	6
Yoshizawa et al. (2020)	+	+	–	+	+	–	?	+	+	+	7
Yoshizawa and Yoshida (2022)	+	+	+	+	+	–	?	+	+	+	8
Chen et al. (2025)	+	+	+	+	+	+	?	+	+	+	9

+, ye; -, no; ?, unable to determine.

**Table 4:** Participant characteristics for the studies included in this review.

Study	Participants information						
	Group	Sex	Age (years)	Height	Body mass (kg)	Sample size (*n*=2275)	Population
Abd El-Kader (2014)	Training	Male	62.2 ± 3.3	168.6±7.4	70.6±4.9	NR	Community-dwelling elder
	Control	Male	60.8 ± 3.7	170.3±6.1	68.9±5.1	NR	
Cattagni et al. (2014)	Healthy young adults	NR	24.1 ± 5.0	180.4 ± 5.9	72.5 ± 7.8	21	Healthy population
	Healthy middle-aged adults	NR	50.2 ± 4.5	174.7 ± 8.8	74.8 ± 16.3	12	
	Healthy elderly non-fallers	NR	75.5 ± 7.0	164.0 ± 7.7	63.6 ± 11.6	27	
	Elderly faller	NR	79.8 ± 6.7	160.4 ± 8.6	63.7 ± 12.4	39	Elderly fallers
Chen et al. (2021)	NA	Male	21 ± 1	180.6 ± 6.0	73.3 ± 9.8	31	Recreationally trained male runners (Tier 1)
Cobb et al. (2014)	NA	Male (n=40) Female (n=68)	22.8 ± 4.7	168.5 ± 10.4	69.9 ± 13.3	108	Healthy population
Ding et al. (2024)	NA	Male (n=15) Female (n=15)	14.5 ± 0.5	178.6 ± 11.1	70.0 ± 15.4	30	Basketball players (Tier 3)
Feehan et al. (2022)	NA	Female	20.6 ± 1.2	163.7 ± 8.4	62.2± 10.16	17	Football players (Tier 2 )
Hagen et al. (2020)	Young adults	Male (n=15) Female (n= 15)	25.0±3.7	181±10	73.6±14.3	61	Physically active population
	Older adults	Male (n=15) Female (n=16)	66.3±5.7	168±7.7	76.3±15.3		
Hébert-Losier et al. (2023)	NA	NA	22.9 ± 2.7	182.6 ± 4.8	93.2 ± 6.3	16	Rugby Players (Tier 2)
Hirono et al. (2020)	NA	Male (n=19) Female (n=14)	23 ± 2	166.6 ± 8.3	60.3 ± 11.7	33	Healthy population
Lin et al. (2009)	NA	Male (n= 14) Female (n = 14)	19.8±1.4	169.9±8.2	65.1± 8.3	28	Healthy population
İnal et al. (2012)	NA	Male (n=6)	21.3 ± 3.4	175.6 ± 5.4	71.5 ± 6.6	11	Elite sprinters
		Female (n=5)		167.8 ± 5.2	57.0 ± 4.9		
Kadlubowski et al. (2024)	NA	Male	16.62 ± 1.1	178 ± 6	67.7 ± 8.5	45	Youth soccer players (Tier 3)
Kayhan et al. (2024)	NA	Male	18.56 ± 0.50	176 ± 8	67.80 ± 1.07	38	Youth soccer players (Tier 4)
Kim and Park (2015)	Defenders (n=7)	Female	NR	NR	NR	18	Elite hockey players (Tier 2)
	Forwards (n=5)						
	Midfielders(n=6)						
Kouzaki and Shinohara (2010)	Young men (n=20)	Male	28.1 ± 4.0	172.9 ± 5.4	69.3 ± 8.3	40	Healthy population
	Elderly men (n=20)		69.7 ± 2.8	165.0 ± 6.6	61.1 ± 7.9		
Kozinc et al. (2021)	Basketball (n= 163)	Male (n=106) Female (n=57)	16.8 ± 1.4	183.9 ± 9.9	76.8 ±13.1	316	Professional athletes (Tier 2)
	Long distance runners (n=49)	Male (n=30) Female (n=19)	32.8 ± 10.2	176.1 ± 10.2	71.5 ± 11.1		
	Tennis (n=104)	Male (n=60) Female (n=42)	16.9 ± 8.6	172.1 ± 10.9	62.9 ± 12.6		
Möck et al. (2018)	NA	Male (n= 40) Female (n= 16)	23.7±3.0	176.9±8.1	74.2±10.3kg	56	Healthy young participation
Möck et al. (2023)	NA	NR	23.7 ± 3.0	176.9 ± 8.1	74.2 ± 10.3	56	Physical education students
Muehlbauer et al. (2013)	NA	NR	23.4 ± 3.9	169.4 ± 7.8	63.9 ± 9.6	27	Healthy young adults
Muehlbauer et al. (2012)	NA	NR	55.8 ± 3.5	174.4 ± 8.2	78.2 ± 10.4	32	Healthy middle-aged adults
Oshita and Yano (2012)	NA	Male	21 ± 1.0	170.6 ± 6.1	65.1 ± 7.2	12	Healthy adults
Ranisavljev et al. (2014)	NA	Male	22.1 ± 1.6	182.6 ± 4.6	78.5 ± 8.4	29	Recreationally active athletes (Tier 1)
Sara et al. (2021)	NA	Male (n=14)	21.5±1.8	181± 8	64.0±10.8	28	Healthy adults
		Female (n=14)	21.1±2.9	166± 7	79.4±10.3		
Shimizu et al. (2024)	NA	Female	20.5±0.9	160.1±5.3	53.4±3.8	26	Healthy adults
Singh et al. (2022)	NA	NR	20.08 ± 1.94	170.67 ± 9.25	63.43 ± 10.23	27	University-level athletes (Tier 2)
Słomka and Michalska (2024)	NA	Male (n=14)	22.3 ± 1.8	181.7 ± 5.1	79.7 ± 10.1	30	Healthy adults
		Female (n=14)	22.3 ± 1.8	164.6 ± 6.5	63.1 ± 10.6		
Genuario and Dolgener (1980)	NA	Female	NR	NR	65.8 ± 8.07	29	Active athletes (Tier 2)
Tao et al. (2020)	NA	Male (n=18) Female (n=22)	23.7 ± 4.9	NR	NR	40	Healthy adults
Trajković et al. (2021)	Basketball	Male (n=157) Female (n=58)	16.7 ± 1.3	183.9 ± 9.8	75.6 ± 12.9	165	Highly trained athletes (Tier 3)
	Dance	Male (n=23) Female (n=54)	22.6 ± 6.6	170.3 ± 7.5	60.0 ± 9.7	77	
	Soccer	Male (n=169) Female (n=0)	17.4 ± 3.4	179.4 ± 7.1	70.2 ± 9.5	169	
	Track and field	Male (n=21) Female (n=8)	17.8 ± 2.7	176.8 ± 8.0	70.1 ± 9.5	29	
	Volleyball	Male (n=42) Female (n=0)	16.9 ± 3.8	183.2 ± 9.3	73.4 ± 12.7	42	
	Alpine skiing	Male (n=9) Female (n=0)	22.7 ± 3.3	181.5 ± 6.9	82.3 ± 5.6	9	
	Tennis	Male (n=68) Female (n=42)	16.9 ± 10.4	172.7 ± 11.0	62.2 ± 12.8	110	
	Martial arts	Male (n=18) Female (n=17)	18.7 ± 4.9	172.5 ± 9.5	68.7 ± 16.0	35	
	Speed Skating	Male (n=12) Female (n=7)	16.6 ± 4.3	166.5 ± 13.8	59.5 ± 14.9	19	
Vecbērza et al. (2025)	NA	Male	20.3 ± 3.0	181.5 ± 8.5	77.4 ± 12.2	32	Floorball players (Tier 3)
Wyrick (1969)	NA	Female	NR	162.4	54.3	56	Recreationally active athletes (Tier 1)
Yoshizawa et al. (2020)	NA	Male (n=30)	21.1 ± 3.8	NR	NR	52	Healthy adults
		Female (n=22)	21.6 ± 4.8	NR	NR		
Yoshizawa and Yoshida (2022)	NA	Male	20.5 ± 3.6		65.3 ± 8.7	21	Healthy adults
Chen et al. (2025)	NA	Male (n=41) Female (n= 16)	17.4 ± 4.5	178.1 ± 9.7	71.9 ± 13.6	57	Soccer and basketball players (Tier 2)

NA, not applicable; NR, not reported.

### Methodological quality and risk of bias

Study methodological quality is presented in **Table 5**. We were able to explicitly confirm internal validity for 32/34 included studies, and external validity was found in 31/34 studies, as the studies failed to report the proportion of participants recruited relative to the sample population. Scores ranged between 6/10 and 10/10 for study methodological quality and risk of bias.

**Table 5:** Characteristics of studies included in this review.

Study	Ankle strength				Physical performances				Associations				
	Test	Reported value	Criterion measure	Test-retest reliability	Physical performance	Reported value	Criterion measure	Reliability of test-retest	Analysis	Variables	r-value	p-value	CI
Abd El-Kader (2014)	DF	CG (18.7±1.6) TG (13.13±1.64)	kg	NR	Berg balance scale	CG (54.4±1.6), TG (48.6±1.7)	Score (0-56)	NR	Pearson correlation	Berg balance test with DF muscle force (CG)	0.95	>0.05	NR
					Functional reach test	CG (46.5±2.0), TG (40.0±3.4)	cm	NR		Functional reach test with DF muscle force ( CG)	0.96	>0.05	NR
					Timed to get up test	CG (9.2±1.2), TG (12.7±2.1)	sec	NR		Timed to get up with DF muscle force (CG)	-0.89	>0.05	NR
										Berg balance test with DF muscle force (training group)	0.94	>0.05	NR
										Functional reach test with DF muscle force( TG)	0.95	>0.05	NR
										Timed to get up with DF muscle force (TG)	-0.88	>0.05	NR
Cattagni et al. (2014)	MIT PF	320.4 ± 64.7 (YA), 235.4 ± 65.8 (MA), 164.6 ± 63.2 (ENF), 105.5 ± 34.8 (EF)	N.m	NR	Normalized CoP	208.8± 61.0 (YA), 271.5±78.3 (MA), 290.4±91.5 (ENF), 576.5±324.5 (EF)	mm, mm2	NR	Linear regression	PF/DF MIT with Normalized CoP (curvilinear)	0.68	0.01	NR
	MIT DF	89.6 ± 13.8 (YA), 75.9 ± 20.2 (MA), 57.7 ± 19.2 (ENF), 42.7 ± 9.9 (EF)								PF/DF MIT with Normalized CoP (linear)	0.6	0.01	NR
	DF nMIT	1.2 ± 0.1 (YA), 1.0 ± 0.2 (MA), 0.9 ± 0.2 (ENF), 0.7 ± 0.2 (EF)								non-fallers PF/DF MIT with Normalized CoP (linear)	0.4	0.01	NR
	PF nMIT	4.4 0.7(YA), 3.2 0.7 (MA), 2.5 0.7(ENF),1.7 0.5 (EF)								elderly fallers PF/DF MIT with Normalized CoP (linear)	0.55	0.01	NR
Chen et al. (2021)	PF	1823.7 ± 341.4	N (60°/s)	NR	leg spring stiffness at 12kmh	12.4 ± 1.5	Km.h-1	0.8 to 0.9 (ICC)	Pearson’s correlation	PTq of PF with leg spring stiffness test	0.561	0.01	NR
	DF concentric	37.0 ± 6.4			leg spring stifness at 14kmh	12.1 ± 1.2				PTq of PF concentric with leg spring stiffness test	0.571	0.012	NR
	PF concentric	128.1 ± 23.7								PTq of DF eccentric with leg spring stiffness test	0.506	0.031	NR
	DF eccentric	59.1 ± 8.3								PTq of PF eccentric with leg spring stiffness test	0.436	0.014	NR
	PF eccentric	223.5 ± 45.2											NR
Cobb et al. (2014)	Isometric PF	0.5 ± 0.1	N.m.	NR	Postural Stability	NR		NR	Multiple regression analysis	DF/PF, IV/EV with mediolateral postural stability	0.098	0.05	NR
	Isometric DF	0.2 ± 0.0								DF/PF, IV/EV with Anteroposterior postural stability	0.042	0.05	NR
	Isometric IV	0.1 ± 0.0											
	Isometric EV	0.1 ± 0.0											
Ding et al. (2024)	PF (dominant)	1.2±0.3	N.m (60°/s )	0.9	CMJ	33.1±4.5	cm	0.9		ankle plantar flexor asymmetry and CMJ	−0.56	0.01	NR
	PF (non-dominant)	0.9±0.3		0.9						PF Asymmetry with CMJ (boys)	-0.47	0.05	NR
	DF (dominant)	0.4±0.1		0.9						PF Asymmetry with CMJ (girls)	-0.78	0.01	NR
	DF (non-dominant)	0.3±0.1	N.m.	0.9									NR
Feehan et al. (2022)	IV PTq Left	15.9 ±10.6	lb/ft	NR	Reaction time left	0.3 ± 0.0	s	NR	Pearson’s correlation	Right EV torque with Right RT	-0.53	0.03	NR
	IV PTq Right	13.8 ±9.4			Reaction time right	0.3 ± 0.0	s	NR		Left IV torque with left RT	-0.24	0.02	NR
	EV PTq Left	12.5 ±7.2											
	IV PTq Right	14.9 ±7.2											
Hagen et al. (2020)	MVIC inversion	16.1±4.2(YA), 9.09±3.42(OA)	Nm	NR	YA LOS A-P	58.1±9.9	%	NR	Pearson’s correlation	Evertor peak torque with AP-LOS (YA)	0.417	0.05	NR
	MVIC eversion	12.7±2.92(YA), 7.6±3.0 (OA)			YA LOS M-L	73.8±16.3	%			Summed muscle strength with AP-LOS (YA)	0.455	0.05	NR
	IV/EV ratio	28.9±5.6 (YA) 16.7±5.54(OA)			YA CoG sway	118.6±29.2	cm			Invertor peak torque with ML-LOS (YA)	0.408	0.05	NR
					YA CoG velocity	0.0±0.0	m/s			Summed muscle strength with ML-LOS (YA)	0.425	0.05	NR
					OA LOS A-P	46.2±8.9	%			Evertor peak torque with AP-LOS (OA)	0.396	0.05	NR
					OA LOS M-L	71.1±14.5	%			Summed muscle strength with AP-LOS (OA)	0.362	0.05	NR
					OA CoG sway	217.7±79.2							
					OA CoG velocity	0.1±0.1	m/s						
Hébert-Losier et al. (2023)	Calf muscle with BW/Power	653.9 ± 93.0	W	ICC ≥0.8, CV ≤ 9%	10m Sprint average	1.6 ± 0.0	s	0.9	Linear regression	Body weight power with average 10m sprint time	-0.531	0.034	-0.807 to 0.031
	Calf muscle with weighted Power	655.6 ± 112.6	W		10m sprint best	1.6 ± 0.0	s	0.9		Weighted power with average 10m sprint time	-0.628	0.009	-0.851 to -0.173
	Strength-endurance (repetitions displacement)	21.1 ± 4.8	n							Strength endurance total displacement with average 10m sprint time	-0.505	0.046	-0.794 to 0.005
	Strength-endurance (peak displacement	10.3 ± 1.9	cm							Strength endurance total work with 10m average sprint time	-0.545	0.029	-0.814 to 0.05
	Strength endurance total displacement	216.7 ± 50.4	cm							Strength endurance with 10m best sprint time	-0.527	0.036	-0.805 to 0.025
	Strength endurance total work	2153.8 ± 497.0	j							Strength endurance peak with 10m best sprint time	-0.512	0.043	-0.797 to 0.004
										Strength endurance total displacement with 10m best sprint time	-0.505	0.046	-0.794 to 0.005
										Strength endurance total with 10m best sprint time	-0.503	0.047	-0.793 to 0.007
Hirono et al. (2020)	Maximal isometric strength of PF	2.3 ± 0.4	Nm	NR	CoP fluctuation on a stable platform	6.5 ± 1.9	cm/(cm·kg) × 10–5	NR	Pearson’s correlation	COP with CV of force at 5% of MVT (stable platform)	0.512	0.002	NR
	CV of force at 5% of PF MVT	1.7 ± 0.5			Cop fluctuation on an unstable platform	4.8 ± 1.5				COP with CV of force at 20% of MVT (unstable platform)	0.458	0.007	NR
	CV of force at 20% of PF MVT	1.3 ± 0.4											
	CV of force at 50% of PF MVT	1.3 ± 0.3											
	1RM calf raise	99.0 ±21.1	kg	0.9									
Lin et al. (2009)	EV D 30°/s	13.7 ± 3.0	Nm/kg	NR	SLB average RD OE D	0.7 ± 0.1	in	NR	Pearson correlation	There was no statistically significant correlations			
	EV ND 30°/s	13.4±2.9			SLB average RD OE ND	0.7±0.1	in						
	IV D 30°/s	14.9±3.1			SLB average RD CE D	1.3±0.2	in						
	IV ND 30°/s	14.7±3.5			SLB average RD CE ND	1.3±0.2	in						
	Ankle IV/EV ratio D 30°/s	0.9±0.1			95% EA OE D	5.6±2.4	in.2						
	Ankle IV/EV ratio D 30°/s	0.9±0.1			95% EA CE ND	5.9±2.6	in.2						
	EV D 120°/s	9.2±1.7			95% EA OE D	23.6±5.2	in.2						
	EV ND 120°/s	9.1±1.5			95% EA OE ND	24.1±5.4	in.2						
	IV D 120°/s	11.1±2.7			SLB average velocity OE D	4.7±0.9	in./s						
	IV ND 120°/s	10.9±2.7			SLB average velocity OE ND	4.7±0.9	in./s						
	Ankle IV/EV ratio D 120°s	0.8±0.1			SLB average velocity CE D	9.8±2.3	in./s						
	Ankle IV/EV ratio ND 120°s	0.8±0.1			SLB average velocity CE ND	9.8±2.4	in./s						
İnal et al. (2012)	DF Power (P) Right	9.8 ± 1.2 (F)	Nm	NR	100m linear sprint	12.2 ± 0.4 (F)	s	NR	Pearson correlation	120°/s right IV average power with 100-m sprint time [M]	-0.866	0.026	NR
		14.6 ± 3.5 Male (M)			100m linear sprint	10.8 ± 0.2 (M)				30°/s right EV average power with 100-m sprint time [M]	-0.82	0.046	NR
	DF P Left	10.2 ± 2.3 (F)								30°/s left EV average power with 100-m sprint time [M]	-0.86	0.028	NR
		14.6 ± 3.5 (M)								120°/s right DF average power with 100-m sprint time [F]	-0.975	0.005	NR
	Plantar flexors Right	18.3 ± 6.8 (F)								120°/s left DF average power with 100-m sprint time [F]	-0.836	0.05	NR
		37.9 ± 6.8 (M)											
	Plantar flexors left	18.5 ± 4.6 (F)											
		38.0 ± 10.5 (M)											
	Invertors right	5.8 ± 0.7 (F)											
		14.7 ± 3.6 (M)											
	Invertors left	5.3 ± 2.0 (F)											
		18.2 ± 11.6 (M)											
	Evertors right	7.20 ± 0.9 (F)											
		13.1 ± 5.6 (M)											
	Evertors left	6.6 ± 1.2 (F)											
		13.6 ± 5.5 (M)											
Kadlubowski et al. (2024)	1RM calf raise	99.0± 21.1	kg	0.9	Linear Sprint- 5m	0.9±0.0	s	0.8	Pearson’s correlation	1RM CR with RSI 45cm	0.358	<0.05	NR
	Relative 1RM CR	1.4±0.3	Kg/kg		Linear Sprint- 10m	1.7±0.0		0.9		1RM CR with RSI 60cm	0.456	<0.05	NR
					Linear Sprint- 30m	4.1±0.1	s	0.9		Relative 1RM CR with RSI 45cm	0.348	<0.05	NR
					CMJ height	41.2±4.5	cm	0.9		Relative 1RM CR with RSI 60cm	0.456	<0.05	NR
					RSI - 15cm	156.8±33.6	cm/s	0.9					
					RSI - 30cm	163.8±39.9	cm/s	0.9					
					RSI - 45cm	163.2±26.8	cm/s	0.8					
					RSI - 60cm	152.5±36.5	cm/s	0.8					
Kayhan et al. (2024)	PF	NR	Kg	NR	dynamic balance	NR	Index (ranging 1 to 5)	NR	Pearson’s correlation And linear regression	DLDF strength with DL balance	-0.382	0.002	NR
	DF	NR	kg	NR	RSI (30cm)	NR	m/s	NR		DL DF strength with DL RSI	0.326	0.043	NR
										NDL DF strength with DL RSI	0.598	0.00	NR
										NDL DF strength with NDL RSI	0.602	0.00	NR
										DL PF strength with DL balance	-0.348	0.03	NR
										NDL PF strength with DL balance	-0.319	0.046	NR
										NDL PF strength with NDL balance	-0.28	0.048	NR
										DL PF strength with NDL RSI	0.309	0.047	NR
										NDL PF strength with DL RSI	0.446	0.004	NR
										NDL PF strength with NDL RSI	0.384	0.016	NR
Kim and Park (2015)	Eccentric EV PTq at 120°/s (FW)	51.9 ± 4.2	Nm	NR	SLS-SI (FW)	2.3 ±0.6	index	NR	Pearson's correlation	FW- Static balance (Lopez et al.) with Eccentric EV PTq at 120°/s	-0.925	<0.05	NR
	Eccentric EV PTq at 90°/s (FW)	37.4 ± 21.0		NR	TCT (FW)	151.8 ±42.1	s	NR		FW-Dynamic balance(Lundberg et al.) with Eccentric EV PTq at 120°/s	-0.91	<0.05	NR
	concentric EV PTq at 60°/s (MF)	34.0± 6.5		NR	SLS-SI (MF)	2.2± 0.8	index	NR		MF- Static balance (Lopez et al.) with concentric EV PTq at 60°/s	0.897	<0.05	NR
	Eccentric EV PTq at 120°/s(MF)	70.8± 32.7		NR	TCT (MF)	134.3± 16.7	s	NR		FW- Static balance (Lopez et al.) with Eccentric EV PTq at 90°/s	-0.892	<0.05	NR
										MF-Dynamic balance (Lundberg et al.) with Eccentric EV PTq at 120°/s	-0.862	<0.05	NR
										MF-Dynamic balance (Lundberg et al.) with Eccentric EV PTq at 120°/s	0.919	<0.01	NR
Kouzaki and Shinohara (2010)	PF (MVC)		%	NR	bipedal quiet standing		mm		Pearson’s correlation	CV of COP with PF EMG power at 10-15 Hz	0.455	<0.01	NR
										CV of COP with CV of PF force at 2.5% of MVC	0.62	<0.001	NR
										CV of COP with PF force at 5% of MVC	0.455	<0.001	NR
Kozinc et al. (2021)	Basket ball				CoD90		s	0.9	Pearson’s correlation	Basketball			
	PF PTq	3.6 ± 0.8		0.9(ICC), 3.0 (CV)	CoD 180		s	0.9		PF PTq with CoD 90	-0.42	<0.01	NR
	PF rate of torque development (RTD) 50	12.8 ± 4.0		0.7 (ICC), 11.3 (CV)						PF RTD 50 with CoD 90	-0.38	<0.01	NR
	PF RTD 100	16.2 ± 4.8		0.7 (ICC), 9.5 (CV)						PF RTD 10 0 with CoD 90	-0.44	<0.01	NR
	DF PTq	1.0 ± 0.2		0.9 (ICC), 2.3 (CV)						PF PTq with CoD 180	-0.38	<0.01	NR
	DF RTD 50	6.1 ± 1.5		0.7 (ICC), 9.2 (CV)						PF RTD 50 with CoD 180	-0.37	<0.01	NR
	DF RTD 100	5.8 ± 1.6		0.7(ICC), 7.0(CV)						PF RTD 10 0 with CoD 180	-0.38	<0.01	NR
	Running									DF PTq with CoD 90	-0.22	<0.01	
	PF PTq	3.4±0.8		0.9(ICC), 2.0(CV)						DF RTD 50 with CoD 90	-0.32	<0.01	NR
	PF rate of torque development (RTD) 50	12.3± 4.3		0.8 (ICC), 8.9 (CV)						DF RTD 10 0 with CoD 90	-0.29	<0.01	NR
	PF RTD 100	15.1 ± 4.6		0.8 (ICC), 7.5 (CV)						DF PTq with CoD 180	-0.29	<0.01	NR
	DF PTq	1.0 ± 0.1		0.9(ICC), 1.9(CV)						DF RTD 50 with CoD 180	-0.41	<0.01	NR
	DF RTD 50	5.9 ± 1.4		0.7 (ICC), 9.9(CV)						DF RTD 10 0 with CoD 180	-0.39	<0.01	NR
	DF RTD 100	5.4±1.1		0.8(ICC), 6.6(CV)						Running			
	Tennis									PF PTq with CoD 90	-0.3	<0.05	NR
	PF PTq	3.6 ± 0.8		0.9(ICC), 2.6 (CV)						PF RTD 10 0 with CoD 90	-0.3	NR	
	PF rate of torque development (RTD) 50	13.1±3.9		0.8 (ICC), 8.4(CV)						Tennis			
	PF RTD 100	16.1 ± 4.5		0.9 (ICC), 6.5(CV)						PF PTq with CoD 90	-0.23	<0.05	NR
	DF PTq	0.9 ± 0.1		0.9(ICC), 3.7(CV)						PF RTD 50 with CoD 90	-0.24	<0.05	NR
	DF RTD 50	5.4 ±1.4		0.8(ICC), 7.7(CV)						PF RTD 10 0 with CoD 90	-0.29	<0.01	NR
	DF RTD 100	5.0 ±1.1		0.9(ICC), 5.7(CV)						PF PTq with CoD 180	-0.39	<0.01	NR
										PF RTD 50 with CoD 180	-0.36	<0.01	NR
										PF RTD 10 0 with CoD 180	-0.37	<0.01	NR
										DF PTq with CoD 90	-0.28	<0.01	NR
										DF RTD 50 with CoD 90	-0.32	<0.01	NR
										DF RTD 10 0 with CoD 90	-0.37	<0.01	NR
										DF PTq with CoD 180	-0.48	<0.01	NR
										DF RTD 50 with CoD 180	-0.39	<0.01	NR
										DF RTD 10 0 with CoD 180	-0.47	<0.01	NR
Möck et al. (2018)	1 RM Calf raise	191.9 ± 44.1	kg	0.9 (p=0.01) test -retest	Sprint test 0-5m	1.0 ± 0.0	s	r = 0.94–0.98 (p < 0.05)	Pearson’s correlation	CR 1 RM with 5m sprint time	-0.483	<0.01	NR
	Relative1RM Calf raise	2.5 ± 0.4	kg/kgBW		5-10m	0.7± 0.0	s			CR 1 RM with 10m sprint time	-0.663	<0.01	NR
					10-15m	0.6± 0.0	s			CR 1 RM with 15m sprint time	-0.657	<0.01	NR
					15-20m	0.6 ± 0.0	s			CR 1 RM with 20m sprint time	-0.741	<0.01	NR
					20-25m	0.6± 0.0	s			CR 1 RM with 25m sprint time	-0.7	<0.01	NR
					25-30m	0.6± 0.0	s			CR 1 RM with 30m sprint time	-0.72	<0.01	NR
										CR 1 RM relative with 5m sprint time	-0.46	<0.01	NR
										CR 1 RM relative with 10m sprint time	-0.541	<0.01	NR
										CR 1 RM relative with 15m sprint time	-0.508	<0.01	NR
										CR 1 RM relative with 20m sprint time	-0.564	<0.01	NR
										CR 1 RM relative with 25m sprint time	-0.545	<0.01	NR
										CR 1 RM relative with 30m sprint time	-0.577	<0.01	NR
Möck et al. (2023)	1 RM Calf raise	191.9 ± 44.1	kg	0.9	SJ	31.9±6.5	cm		Pearson’s correlation	CR 1 RM with SJ	0.659	<0.01	0.479–0.786
	Relative1RM Calf raise	2.5± 0.4	kg/kgBW		CMJ	34.2 ±7.4	cm			CR 1 RM with CMJ	0.708	<0.01	0.547–0.819
					RSI 16	155.1±36.7	m/s			CR 1 RM with RSI 16 cm	0.326	<0.01	0.110–0.571
					RSI 24	162.6±35.9	m/s			CR 1 RM with 24 cm	379	<0.01	0.129–0.584
					RSI 32	168.2±36.2	m/s			CR 1 RM with 32 cm	526	<0.01	0.305–0.693
					RSI 40	166.8±38.9	m/s			CR 1 RM with 40 cm	514	<0.01	0.290–0.684
					RSI 48	163±38.95	m/s			CR 1 RM with 48 cm	457	<0.01	0.221–0.643
										CR 1 RM relative with SJ	575	<0.01	0.368–0.728
										CR 1 RM relative with CMJ	565	<0.01	0.355–0.721
										CR 1 RM with relative RSI 16 cm	436	<0.01	0.196–0.627
										CR 1 RM relative with 24 cm	472	<0.01	0.239–0.654
										CR 1 RM relative with 32 cm	573	<0.01	0.365–0.726
										CR 1 RM relative with 40 cm	535	<0.01	0.317–0.700
										CR 1 RM relative with 48 cm	521	<0.01	0.299–0.689
Muehlbauer et al. (2013)	Maximal isometric torque of PF	133.0 ± 39.7	N.m	0.9	CoP (AP)	639.5 ± 129.0	mm	0.8	Pearson correlation	PF MIT with CMJ P	0.458	<0.05	NR
	RTD	492.9 ± 182.0	N.m.s	0.9	CoP (ML)	739.3 ± 175.5	mm	0.8		PF MIT with CMJ H	0.511	<0.01	NR
					CoP (AP)	804.9 ±260.5	mm	0.8		PF RTD with CMJ P	0.689	<0.01	NR
					CoP (ML)	796.0 ± 268.0	mm	0.8		PF RTD with CMJ H	0.628	<0.01	NR
					CMJ Power	25.2 ± 4.4	cm	0.9					
					CMJ height	40.9 ± 6.8	W.kg	0.9					
Muehlbauer et al. (2012)	MIT of PF	151.7 ± 42.6	N.m	0.9	Static balance CoP (AP)	1,159.2 ± 510.8	mm	0.8	Pearson’s correlation	PF MIT with CMJ power	0.482	<0.001	NR
	RTD	492.5 ± 206.1	N.m.s	0.9	Static balance CoP (ML)	1,113.2 ± 273.7	mm	0.8		PF MIT with CMJ height	0.501	<0.001	NR
					Dynamic balance CoP (AP)	497. 9 ± 221.3	mm	0.8		PF RTD with CMJ power	0.399	<0.005	NR
					Dynamic balance CoP (ML)	801.8 ± 487.3	mm	0.8		PF RTD with CMJ height	0.361	<0.005	NR
					CMJ Power	20.3 ± 3.5	cm	0.9					
					CMJ height	31.4 ± 5.6	W.kg	0.9					
Oshita and Yano (2012)	PF force fluctuation at 10% MVC	0.5 ± 0.1	N	NR	Postural sway (A-P)	0.6 ± 0.1	cm/	NR		PF force fluctuation at 10% MVC with Postural sway	0.58	0.05	NR
	PF force fluctuation at 20% MVC	0.9 ± 0.1	N	NR									
Ranisavljev et al. (2014)	PF Isometric	16.5±2.6	Fmax(Nkg 2/3)	NR	walk-to-run speed (WRT)	8.0 ± .2	km/h			DF Isometric with WRT	0.37	<0.05	NR
	DF Isometric	68.6±13.2	Fmax(Nkg 2/3)		run-to-walk speed (RWT)	7.6±.3	km/h			PF Isokinetic60/s with WRT	0.365	<0.05	NR
	PF Isokinetic60/s	0.5±0.1	Torque(Nmkg -1)							DF Isometric with RWT	0.392	<0.05	NR
	DF Isokinetic60/s	1.6±0.3	Torque(Nmkg -1)										
	PF Isokinetic180/s	0.4±0.1	Torque(Nmkg -1)										
	DF Isokinetic180/s	1.0±0.2	Torque(Nmkg -1)										
Sara et al. (2021)	PF MVIC PTq (M)	148.8±36.3	Nm	NR	Single-leg heel raise task to failure (M)	32.6±6.9	Reps		Pearson’s correlation	There are no significant correlation			
	PF MVIC PTq (F)	128.1±34.1	Nm		Single-leg heel raise task to failure (F)	39.4±14.8	Reps						
Shimizu et al. (2024)	PF torque (60 deg/s)	0.4±0.2	Nm/kg	NR	CMJ height	NR	m	NR	Pearson’s correlation	There are no significant correlation			
					Rebound drop jump height	0.3 ± 0.0	m	NR					
					Contact time (1st half)	0.1 ± 0.0	s	NR					
					Contact time (2nd half)	0.2 ± 0.0	s	NR					
					Contact time (total)	0.4 ± 0.1	s	NR					
					RDJ-index	0.8 ± 0.3	m/s	NR					
Singh et al. (2022)	DL PF (30°/s)	39.6±8.7	Ft/lbs	NR	single-leg hop jump Power - DL	9.5±2.8	kcal/s	NR		DL PF (30°/s) with DL power	0.83	< 0.001	NR
	DL DF (30°/s)	33.1±5.2			single-leg hop jump Power - NDL	8.4±5.4	kcal/s	NR		DL DF (30°/s) with DL power	0.56	< 0.001	NR
	DL PF (120°/s)	37.3±12.2								DL PF (120°/s) with DL power	0.85	< 0.001	NR
	DL DF (120°/s)	37.5±12.0								DL DF (120°/s) with DL power	0.73	< 0.001	NR
	NDL PF (30°/s)	26.1±11.9								NDL PF (30°/s) with NDL power	0.61	0.001	NR
	NDL DF (30°/s)	16.9±5.0								NDL PF (120°/s) with NDL power	0.56	0.002	NR
	NDL PF (120°/s)	19.7±15.5											
	NDL DF (120°/s)	17.6±7.4											
Słomka and Michalska (2024)	Left ankle DF		N	ICC = 0.9 (0.95–0.98)	Maximal forward CoP displacement	NR	NR	NR	Pearson’s correlation	CoP D with left PF	0.5	< 0.05	NR
	Left ankle PF		N	ICC = 0.95 (0.97–0.99)	Forward functional stability index	NR	NR	NR		CoP D with right DF	0.41	< 0.05	NR
	Right ankle DF		N	ICC = 0.95 (0.91–0.98)						CoP D with right PF	0.45	< 0.05	NR
	Right ankle PF		N	ICC = 0.9 ( 0.8–0.97)									
Genuario and Dolgener (1980)	PF Fast Tq (180 d/s)	27.5 ± 9.7	Ft-lbs	NR	Vertical jump height	9.8 ± 2.1	In	NR	Pearson’s correlation	Relative vertical jump with PF (slow Tq)	0.424	< 0.05	NR
	PF slow Tq (30d/s)	52.6 ± 14.7	Ft-lbs	NR	Relative vertical jump	118.3 ± 32.9	Ft/lbs	NR		Relative vertical jump with PF (fast Tq)	0.502	< 0.01	NR
Tao et al. (2020)	IV	0.3 ± 0.0	Nm/kg	ICC = 0.8; 95% CI 0.7-0.8	SLB test on stable surface	2.2 ± 1.5	frequency	ICC = 0.5; 95% CI: 0.3-0.6	Pearson’s correlation	There were no significant correlations			
	EV	0.3 ± 0.0	Nm/kg	ICC = 0.8; 95% CI 0.7-0.8	SLB test on unstable surface	6.9 ± 1.7	frequency	ICC = 0.73; 95% CI: 0.6-0.8					
					composite mBESS scores	9.1 ± 2.9	frequency	ICC = 0.7; 95% CI: 0.5-0.8					
Trajković et al. (2021)	PF		Nm	NR	CoP velocity (single leg stance)		mm/s	NR		PF and CoP Total	0.14	< 0.01	NR
	DF		Nm	NR	CoP amplitude		mm	NR		DF and CoP Total	0.07	< 0.05	NR
					CoP frequency		Hz	NR		PF and CoP velocity anterior posterior	0.17	< 0.01	NR
										PF and CoP velocity medial–lateral	0.1	< 0.01	NR
										DF and CoP velocity medial–lateral	0.08	< 0.05	NR
										PF and CoP amplitude AP	0.16	< 0.01	NR
										PF and COP amplitude ML	0.07	< 0.05	NR
										PF and CoP frequency AP	0.16	< 0.01	NR
										PF and COP amplitude ML	0.07	< 0.05	NR
Vecbērza et al. (2025)	Absolute PF strength	151.7 ±17.4	kgF	NR	20 m sprint time	3.04 ±0.1	s	NR	Pearson’s correlation	Relative PF with 20m linear sprint	-0.36	<0.05	NR
	Relative PF strength	1.9 ±0.3	kgF/kg	NR	Unilateral reactive strength index	63.9±12.1	cm/s	NR		Relative PF with unilateral RSI	0.35	<0.05	NR
Wyrick (1969)	DF	48.3 ± 10.5	lbs	NR	Balance - bass stick test (static)	20.5 ± 14.4	score	NR	Pearson’s correlation	PF and bass stick balance	-0.2	<0.01	NR
	PF	146.8 ± 28.3	lbs	NR	Bass stick with no visual cues	56 ± 16.3	score	NR		PF and bass stick balance without visual cues	-0.23	<0.01	NR
					High bass stick(dynamic)	25.6 ± 19.5	score	NR		PF and high bass stick balance	-0.14	<0.01	NR
										DF and bass stick balance	-0.11	<0.01	NR
										DF and bass stick balance without visual cues	-0.09	<0.01	NR
										DF and high bass stick balance	-0.09	<0.01	NR
Yoshizawa et al. (2020)	PF Strength Right leg (M)	3.7 ± 0.7	N/kg	NR	Leg extension torque Right leg (M)	2.4 ± 0.5	Nm/kg	NR		Right leg PF strength (M) with right Leg extensor torque	0.41	<0.05	NR
	PF Strength Left leg (M)	3.9 ± 0.9	N/kg	NR	Leg extension torque Left leg (M)	2.4 ± 0.4	Nm/kg	NR		Left leg PF strength (M) with left Leg extensor torque	0.45	<0.05	NR
	PF Strength Right leg (F)	3.3 ± 1.1	N/kg	NR	Leg extension torque Right leg(F)	1.9 ± 0.3	Nm/kg	NR		Right leg PF strength(F) with right Leg extensor torque	0.8	<0.01	NR
	PF Strength Left leg (F)	3.4 ± 1.2	N/kg	NR	Leg extension torque Left leg (M)	1.9 ± 0.3	Nm/kg	NR		Left leg PF strength (F) with left Leg extensor torque	0.65	<0.01	NR
Yoshizawa and Yoshida (2022)	PF	3.8 ± 0.7	N/kg		Total trajectory length (single leg stance) DL	71.4 ± 21.9	cm	NR		Total trajectory length and PF	−0.41	<0.05	NR
					Outer peripheral area -DL	3.1 ± 1.4	cm2	NR		Outer peripheral area and PF	−0.55	<0.05	NR
Chen et al. (2025)	Conc. 60°/s PF Torque - DL	1.14 ± 0.3	N.m/kg	NR	Horizontal deceleration ability (5 m)	−3.9 ± 0.6	m/s2	NR	Pearson’s correlation	Con60 PTq-PF-NDL with 5m Average deceleration	−0.52	<0.001	−0.71 to −0.29
	Conc. 60°/s PF Torque - NDL	1.18 ± 0.3	N.m/kg	NR						Con60 PTq-PF-DL with 5m Average deceleration	−0.53	<0.001	−0.70 to −0.33

NR, Not reported; CI, Confidence interval; CG, Control group; TG, Training group; DF, Dorsiflexion; PF-Plantarflexion; IV, Inversion; EV, Eversion; MIT, Maximal isometric torque; COP, Center of pressure; YA, Young adults; MA, middle aged; ENF-Elderly non-fallers; EF-elderly fallers; PTq, Peak torque; CMJ, Countermovement jump; SJ, Squat Jump; nMIT, normalized maximal isometric torque; ICC, Intraclass correlation; MVIC, maximal voluntary isometric contraction; LOS, limits of stability; AP, Anteroposterior; ML, Mediolateral; CoG, Center of gravity; OA, Older adults; BW, Body weight; CV-Coefficient of variation; MVT, maximal voluntary contraction; D, Dominant; ND-Non-dominant; SLB, Single leg balance; OE, Open eyes; CE-closed eyes; RM, Repetition maximum; RSI, Reactive strength index; FW, Forward; MF, Midfielder; RTD, Rate of torque development; CR-Calf raise,WRT, Walk to run; RWT, Run to walk; RDJ, Rebound drop jump; M-Male; F, Female.

## Discussion

This systematic review aimed to investigate the relationship between ankle strength variables (e.g., PF, DF, IV, and EV) and physical performance in healthy individuals. This included both health-related fitness components (such as muscular endurance, strength, and flexibility) and skill- and performance-related fitness components (such as speed, vertical jump, and CODS). Among the 34 eligible studies, eleven examined the association between ankle PF strength and static balance, while five focused on the relationship between ankle strength and dynamic balance. Eight studies assessed the connection with jump performance, six with sprint performance, and four with RSI. Additionally, one study explored the association between PF strength and variables such as deceleration, reaction time, muscular endurance, and CODS. Finally, two studies investigated the relationship between ankle PF strength and maximal strength performance.

### Static balance

Across 11 studies, PF strength showed a generally weak-to-moderate association with static balance, with most studies reporting reduced postural sway with greater PF strength (Kouzaki and Shinohara, 2010; Oshita and Yano, 2012; Cattagni et al., 2014; Cobb et al., 2014; Hirono et al., 2020; Trajković et al., 2021; Yoshizawa and Yoshida, 2022), although several found no association (Wyrick, 1969; Muehlbauer et al., 2012; Muehlbauer et al., 2013; Słomka and Michalska, 2024). This suggests that PF strength may contribute to postural control but is not a primary determinant, with variability likely influenced by differences in testing methods and populations. While improved corrective ankle torque may explain these findings (Han et al., 2014; Kim and Hwang, 2018), such mechanisms were not directly assessed and should be interpreted cautiously.

Evidence for DF strength is less consistent, with most studies reporting no significant association (Wyrick, 1969; Cattagni et al., 2014; Cobb et al., 2014), and some showing variable relationships depending on measurement approach (Kim and Park, 2015; Trajković et al., 2021). This indicates a limited or context-specific role of DF strength, potentially related to anteroposterior control (Vidal et al., 2023), although this remains speculative.

For IV and EV strength, several studies suggest associations with mediolateral stability (Cobb et al., 2014; Kim and Park, 2015; Tao et al., 2020), but findings are not uniform (Lin et al., 2009; Tao et al., 2020). Differences in assessment conditions, particularly non-weight-bearing strength measures, may partly explain these inconsistencies (Emery, 2003; Mccurdy and Langford, 2006). There is also some indication that strength asymmetry, rather than absolute strength, may be relevant for mediolateral control (Lin et al., 2009).

Lastly, static balance appears to be a multifactorial capacity that cannot be explained by ankle strength alone. Although ankle musculature may contribute to postural stability, balance performance likely reflects integrated neuromuscular control processes (Blackburn et al., 2000; Peterka, 2002; Ivanenko and Gurfinkel, 2018), which were not directly evaluated in the included studies.

### Dynamic balance

Across five studies, PF strength showed no consistent association with dynamic balance, with most studies reporting non-significant relationships across different populations (Wyrick, 1969; Muehlbauer et al., 2012; Muehlbauer et al., 2013; Słomka and Michalska, 2024). Only one study reported a weak-to-moderate negative association in the dominant limb (Kayhan et al., 2024). Overall, these findings suggest that PF strength has a limited role in dynamic balance performance, which is likely influenced by multiple interacting systems rather than isolated ankle strength (Tavakkoli Oskouei et al., 2021). Variability in outcomes may also reflect differences in assessment methods and task demands, as well as advancements in measurement approaches across studies (Takakusaki, 2017; Ivanenko and Gurfinkel, 2018).

In contrast, evidence for DF strength is somewhat more consistent, with several studies reporting significant negative associations with dynamic balance (Abd El-Kader, 2014; Kim and Park, 2015; Kayhan et al., 2024), although others found no relationship (Wyrick, 1969; Słomka and Michalska, 2024). This suggests that DF strength may contribute to dynamic stability in certain contexts, potentially depending on task characteristics and measurement approaches. While it is plausible that dorsiflexors assist in controlling foot placement and center of mass during movement (Almansoof et al., 2023), this mechanism was not directly assessed and should be interpreted cautiously.

For EV strength, limited evidence suggests a potential association with dynamic balance, particularly in athletic populations with high change-of-direction demands (Kim and Park, 2015). However, findings also indicate that ankle muscle ratios and combined strength measures may better predict dynamic balance than isolated strength alone (Hagen et al., 2020), highlighting the importance of considering muscle coordination rather than single muscle groups.

Evidence for IV strength is currently limited to a single study, which reported a moderate association with mediolateral stability in young adults but not in older adults (Hagen et al., 2020). This age-related difference may reflect broader declines in neuromuscular function (Jeon et al., 2023), although such mechanisms were not directly evaluated. More broadly, IV and EV muscles may contribute to lateral stability and foot placement control (Van Leeuwen et al., 2021), but when their contribution is insufficient, compensatory strategies involving proximal segments may be adopted (Donath et al., 2015).

Therefore, the evidence indicates that dynamic balance is not strongly determined by isolated ankle strength. Instead, performance likely reflects task-specific and integrated neuromuscular control processes, with variability across studies largely influenced by differences in assessment methods and participant characteristics.

### Jump

Five studies examined the relationship between ankle PF strength and CMJ height, with findings showing inconsistent but generally positive associations (Muehlbauer et al., 2012; Muehlbauer et al., 2013; Möck et al., 2023; Kadlubowski et al., 2024; Shimizu et al., 2024). Several studies reported moderate-to-strong correlations in healthy populations (Muehlbauer et al., 2012; Muehlbauer et al., 2013; Möck et al., 2023), whereas others found no significant relationship in athletic or specific cohorts (Kadlubowski et al., 2024; Shimizu et al., 2024). These discrepancies suggest that the contribution of PF strength to CMJ performance may be population- and context-dependent. In addition, inter-limb asymmetry in PF strength has been negatively associated with CMJ height, indicating that both strength and symmetry may influence performance (Ding et al., 2024). PF strength may contribute to CMJ performance through its role in force production and transmission during the propulsive phase (Ding et al., 2024). However, CMJ performance is influenced by multiple factors, including neuromuscular coordination and tendon mechanical properties (Li et al., 2021; Wdowski et al., 2023). While stronger PF muscles may enhance force transfer within the kinetic chain (Möck et al., 2023) and influence muscle–tendon unit behavior (Kubo et al., 2021), these mechanisms were not directly assessed in the included studies and should be interpreted cautiously.

Evidence for other vertical jump tasks is limited. Only one study reported a significant positive association between PF strength and SJ performance (Möck et al., 2023), suggesting a potential role of PF strength in concentric-only force production (Donahue et al., 2021), although further research is required. Similarly, evidence for DJ performance is restricted to a single study, which found no significant correlation between ankle strength and jump height, despite differences in PF strength between higher and lower performers (Shimizu et al., 2024). Additional evidence indicates that relative PF strength may be more relevant than absolute strength. An earlier study reported significant associations between normalized PF torque and vertical jump height, despite no relationship with absolute performance (Genuario and Dolgener, 1980). However, these findings should be interpreted cautiously given methodological advancements since that time.

For horizontal jumping, one study reported strong associations between PF strength and single-leg hop performance, with moderate associations for dorsiflexor (DF) strength (Singh et al., 2022). These relationships were stronger in the dominant limb, suggesting a potential influence of limb-specific strength on performance.

The available evidence suggests that ankle PF strength contributes to jump performance, particularly in CMJ and horizontal tasks, but is not a sole determinant. Performance outcomes likely reflect an interaction of strength, coordination, and task-specific mechanical factors, with variability across studies influenced by differences in populations and assessment methods.

### Reactive strength index

Four studies examined the relationship between ankle strength and RSI, generally reporting moderate positive associations (r = 0.32–0.60) (Möck et al., 2023; Kadlubowski et al., 2024; Kayhan et al., 2024; Vecbērza et al., 2025). Both PF and DF strength were positively associated with RSI across different populations and testing protocols, including unilateral repeated jumps and drop jumps. These findings suggest that ankle strength contributes to fast SSC performance, although the magnitude of this relationship varies depending on task characteristics and limb involvement.

Some evidence indicates that non-dominant limb strength may show stronger or more consistent associations with RSI, particularly in athletic populations (Kayhan et al., 2024). This may reflect task-specific roles of each limb during dynamic activities; however, such interpretations remain speculative, as limb function and neuromuscular coordination were not directly assessed. Observed contralateral relationships between limbs further suggest potential interactions in bilateral performance, although the mechanisms underlying these associations are unclear.

The association between PF strength and RSI also appears to be influenced by drop jump height. Both Möck et al. (2023) and Kadlubowski et al. (2024) reported stronger correlations at higher drop heights, whereas relationships were weaker or non-significant at lower heights. This pattern suggests that ankle strength may become more relevant under conditions of increased mechanical demand. While it is plausible that greater PF strength enhances the capacity to tolerate eccentric loading and contribute to subsequent force production (Ishikawa and Komi, 2004; Kubo et al., 2021; Kadlubowski et al., 2024), these mechanisms were not directly evaluated in the included studies.

### Sprint

Six studies examined the relationship between ankle strength and sprint performance across distances ranging from 10 m to 100 m, as well as gait transition speed (İnal et al., 2012; Ranisavljev et al., 2014; Möck et al., 2018; Hébert-Losier et al., 2023; Kadlubowski et al., 2024; Vecbērza et al., 2025). Overall, stronger and more consistent evidence was observed for PF strength during short-distance sprinting, with several studies reporting moderate-to-large negative correlations between PF strength or power and sprint time (Möck et al., 2018; Hébert-Losier et al., 2023; Vecbērza et al., 2025). However, other studies found no significant associations, particularly in elite or youth athlete populations (İnal et al., 2012; Kadlubowski et al., 2024), suggesting that the contribution of PF strength may be context-dependent.

Evidence for DF, IV, and EV strength is more limited but indicates potential associations with sprint performance. Very high correlations have been reported in small samples of elite sprinters (İnal et al., 2012), although these findings should be interpreted cautiously due to limited sample sizes. These results suggest that ankle musculature beyond PF may contribute to sprint performance, but the strength and consistency of these relationships remain unclear.

The stronger associations observed for PF strength during shorter sprint distances may reflect the mechanical demands of the acceleration phase, where rapid force production and short ground contact times are required (Möck et al., 2018; Hébert-Losier et al., 2023; Vecbērza et al., 2025). While it is plausible that PF strength contributes to horizontal force production during early acceleration (Crotty et al., 2024), such mechanisms were not directly assessed in the included studies. In contrast, the contribution of ankle strength to maximal velocity phases appears less consistent, potentially reflecting greater reliance on proximal musculature and elastic mechanisms (Mann and Herman, 1985; Healy et al., 2022).

Indirect evidence further supports a role for ankle musculature in locomotor tasks. Moderate associations have been reported between PF and DF strength and gait transition speed (Ranisavljev et al., 2014), as well as leg stiffness during submaximal running (Chen et al., 2021). These findings suggest a broader contribution of ankle strength to running mechanics, although they do not directly establish relationships with sprint performance. Methodological factors, including the type of strength assessment used (e.g., calf raise vs. dynamic measures), may also influence observed associations (Bolger et al., 2015; Kadlubowski et al., 2024).

Therefore, ankle PF strength appears to be associated with short-distance sprint performance, particularly during acceleration, whereas evidence for other muscle groups and longer sprint distances remains limited and inconsistent. Sprint performance likely reflects an interaction of strength, coordination, and task-specific mechanical factors rather than isolated ankle strength alone (Hamner et al., 2010; Morin et al., 2011; Yüksel et al., 2011).

### Reaction time

Only one study examined the relationship between ankle strength and foot reaction time (Feehan et al., 2022). The authors reported a significant moderate-to-high negative correlation between non-dominant EV strength and contralateral foot reaction time in female collegiate soccer players, with EV strength explaining a portion of the variance in performance (*r* = –0.53; R² = 0.281, *p* = 0.01). These findings suggest a potential association between ankle EV strength and reaction-based motor performance. However, the observed contralateral relationship and underlying mechanisms were not directly assessed and should be interpreted cautiously. Given that evidence is limited to a single study, further research is required before drawing firm conclusions regarding the role of ankle strength in reaction time tasks.

### Deceleration

Only one study examined the relationship between ankle strength and deceleration ability (Chen et al., 2025). The authors reported significant moderate negative correlations between PF strength (both dominant and non-dominant limbs) and short-distance (5 m) deceleration performance in young team-sport athletes (*r* = –0.52 to –0.53, *p* < 0.001). However, no significant association was observed for deceleration following a 10 m sprint.

These findings suggest that PF strength may contribute to deceleration performance under lower-velocity conditions, whereas its influence appears reduced at higher approach speeds (Chen et al., 2025). Although it is plausible that different muscle groups contribute variably across deceleration phases (Haralabidis et al., 2022; Harper et al., 2022; Zhang et al., 2022), these mechanisms were not directly assessed and should be interpreted cautiously. Given that evidence is limited to a single study, further research is required to clarify the role of ankle strength in deceleration tasks.

### Change of direction

Only one study examined the relationship between ankle strength and CODS performance (Kozinc et al., 2021). The authors reported small-to-moderate negative correlations between PF and DF strength and CODS performance across trained runners, basketball players, and tennis players (*r* = –0.22 to –0.49, *p* < 0.01). Basketball and tennis players demonstrated slightly better CODS performance than runners, particularly in 90-degree turns, whereas differences were smaller in 180-degree tasks. These findings suggest a limited association between ankle strength and CODS performance, with potential variability across movement tasks and athlete groups. While sport-specific demands such as frequent cutting and multidirectional movements may influence these relationships (Ujaković and Šarabon, 2020; Kozinc et al., 2021), such factors were not directly evaluated and should be interpreted cautiously. Therefore, CODS performance likely reflects a combination of strength, coordination, and technique rather than isolated ankle strength alone.

### Muscular endurance

Only one study examined the relationship between PF strength and PF endurance (Sara et al., 2021). The authors reported no significant association between maximal isometric PF strength and single-leg heel raise performance, indicating that this test may not be a valid proxy for maximal ankle strength. Similar findings have been reported in clinical populations, where heel raise performance was also not associated with maximal PF strength (Harris-Love et al., 2014). These findings likely reflect the distinct physiological and functional characteristics of strength and endurance measures (Akagi et al., 2008; Rockenfeller et al., 2024). Lastly, the evidence suggests that single-leg heel raise tests should be interpreted as measures of muscular endurance rather than maximal strength.

### Strength

Only one study examined the relationship between PF strength and leg extensor torque (Yoshizawa et al., 2020). The authors reported moderate-to-strong positive correlations between PF strength and leg extensor torque in both males and females (*r* = 0.41–0.80, *p* < 0.05), suggesting a potential association between ankle strength and lower-limb force production. While this relationship may reflect the functional involvement of the calf muscles in multi-joint force generation (Manal et al., 2012; Suzuki et al., 2014; Mengarelli et al., 2018), the underlying mechanisms were not directly assessed and should be interpreted cautiously. These findings indicate a possible synergistic role of ankle musculature in contributing to lower-limb strength, although further research is needed to confirm this relationship.

### Limitations

This review provides a comprehensive examination of the association between ankle strength and physical performance outcomes; however, several limitations should be acknowledged. (a) While this review focused on healthy participants, considerable clinical and methodological heterogeneity prevented the meta-analysis and limited the pooled effect estimations; (b) Variability in factors such as participants’ age, sex, training status, and sporting background may have influenced study outcomes and limited the precision and generalizability of the findings; (c) Methodological inconsistencies further complicated comparisons due to differences in the equipment used and the approaches adopted to assess both independent and dependent variables that introduced potential sources of bias; (d) In relation to balance performance specifically, variations in testing protocols such as eyes open compared with eyes closed conditions or the application of different isokinetic angular velocities likely contributed to discrepancies in reported outcomes; (e) In addition, the small number of available studies addressing certain physical performance domains such as sprinting, jumping, and CODS restricts the strength of the conclusions that can be drawn; (f) The present review did not formally assess publication bias and GRADE certainty of the evidence framework therefore overall robustness of the evidence could not be systematically assessed; (g) Data extraction was performed by one author and verified by a second author rather than through fully independent duplicate extraction, which may have increased the potential risk of extraction bias; (h) The literature search was restricted to the databases available to the authors at the time of the search (i.e., PubMed, Scopus and Web of Science). The absence of additional subject-specific databases such as SPORTDiscus, CINAHL, Embase, and Cochrane Library may be considered a limitation of the study; (i) Lastly, despite the language restriction, no eligible non-English studies were identified, and potential language bias cannot be entirely excluded.

## Conclusion

This systematic review demonstrates a consistent and meaningful association between ankle muscle strength and several key physical performance outcomes in healthy individuals. Greater ankle strength was positively correlated with enhanced vertical jump height, sprint speed, CODS, and RSI and inversely related to postural sway during both static and dynamic balance tasks. These findings highlight the integral role of ankle musculature in supporting components of both health-related and skill-related fitness.

In practical terms, incorporating targeted ankle-strengthening exercises into training programs may contribute to improvements in athletic performance and injury prevention. However, it is important to interpret these associations cautiously, as correlation does not establish causation. To strengthen the evidence base, future research should prioritize longitudinal and interventional study designs and explore differential responses across specific populations, such as youth athletes and elite performers.

## Data Availability

The original contributions presented in the study are included in the article/**Supplementary Material**. Further inquiries can be directed to the corresponding author.
