# Supplementary material for: Associations between ankle strength and physical performance in healthy individuals: a systematic review
Source: Front Physiol. 2026 Jul 15;17:1863201. doi: 10.3389/fphys.2026.1863201 (PMC13413676; doi:10.3389/fphys.2026.1863201)
Supplement: Supplementary file 1 [file SupplementaryFile1.docx]

| **Supplementary file1.** Questions from the modified checklist (Downs & Black, 1998) used to evaluate methodological quality of the included articles. | |
| --- | --- |
| **Question no.** | **Questions** |
|  | **Reporting** |
| 1 | Is the hypothesis/aim/objective of the study clearly described? |
| 2 | Are the main outcomes to be measured clearly described in the introduction or methods section?  **Information outlined in introduction/methodology for both ankle strength and physical performance variables used for associative analysis pertaining to test(s) used, calculation method, and units of measurement* |
| 3 | Are the characteristics of the subjects included in the study clearly described?  **Source defined, with characteristics included* |
| 4 | Are the main findings of the study clearly described? |
| 5 | Does the study provide estimates of the random variability in the data for the main outcomes?  **One of: mean ± SD^a^, standard error^a^, confidence intervals^a^, or interquartile range^b^ outlined for both ankle strength and physical performance variables used for associative analysis* |
| 6 | Have actual probability values been reported (e.g., 0.035 rather than < 0.05) for the main outcomes except where the probability value is < 0.001?  **Exact correlation (r) and significance (p) values provided, specific to the associative analysis* |
|  | **External validity** |
| 7 | Were the subjects asked to participate in the study representative of the entire population from which they were recruited?  **Proportion of subjects asked to participate, relative to the sample population, explicitly stated. Unless evident, then answer "unable to determine"* |
|  | **Internal validity bias** |
| 8 | If any of the results of the study were based on ‘data dredging,’ was this made clear?  **If no signs of retrospective/unplanned data analysis, then answer "yes"* |
| 9 | Were the statistical tests used to assess the main outcomes appropriate? |
| 10 | Were the main outcome measures accurate (valid and reliable)? |
| ^a^Normally distributed data  ^b^Non-normally distributed data | |
