# Supplementary material for: Associations between ankle strength and physical performance in healthy individuals: a systematic review
Source: Front Physiol. 2026 Jul 15;17:1863201. doi: 10.3389/fphys.2026.1863201 (PMC13413676; doi:10.3389/fphys.2026.1863201)
Supplement: Supplementary file 2 [file SupplementaryFile2.docx]

| **Supplementary file 2.** Equipment and manufacturer details of the studies included in this study | | | | |
| --- | --- | --- | --- | --- |
| **Study** | **Ankle strength** | | **Physical performance** | |
|  | **Test** | **Equipment/Manufacturer** | **Test** | **Equipment/Manufacturer** |
| Abd El-Kader (2014) | DF | Handheld dynamometer (Penny and Giles, Biometrics  Division, Blackwood Gwent NP2 IYD United Kingdom) | Berg balance scale | NA |
|  |  |  | Functional reach test | NA |
|  |  |  | Timed to get up test | NA |
| Cattagni et al. (2014) | Isometric PF/DF | Ergometer (I.U.T. Génie Mécanique,  Dijon, France) | Postural stability | Force-platform (Stabilotest, Techno Concept, Cereste, France) |
| Chen et al. (2021) | PF/DF concentric | Motor-driven dynamometer (D&R Ferstl GmbH, Hemau, Germany) | Leg spring stiffness | Visual 3D (v5, C-Motion, Inc., Germantown, MD, United States) |
|  | PF/DF eccentric |  |  |  |
| Cobb et al. (2014) | Isometric PF/DF | Isokinetic dynamometer (Biodex Medical  Systems, Shirley, NY) | Postural Stability | Force platform (model OR6-7-2000; Advanced Mechanical Technologies Inc, Watertown, MA |
| Ding et al. (2024) | PD/DF | IsoMed-2000 isokinetic dynamometer | CMJ | MY JUMP 2 app |
| Feehan et al. (2022) | IV/EV | Biodex III Isokinetic Dynamometer (Biodex Medical Systems, Shirley, NY) | Reaction time | 3 x 4 Switch mats (model 63515A) |
| Hagen et al. (2020) | Isometric IV/EV | Force transducers (Kistler 9321A, Winterthur, Switzerland) | Loss of stability | Force plate (Kistler 9285,  Winterthur, Switzerland) |
|  |  |  | Postural stability |  |
| Hébert-Losier et al. (2023) | Calf raise | iOS Calf Raise application version 1.5.1 | 10m sprint | Single-beam timing lights (Brower Timing System, Utah, USA). |
| Hirono et al. (2020) | Isometric PF | Biodex System 4 (Biodex Medical Systems, Shirley, NY, USA) | Single leg stance | Biodex Balance System SD (Biodex  Medical Systems, Shirley, NY, USA) |
| Lin et al. (2009) | IV/EV | Biodex System 3 Dynamometer and Biodex  Advantage Software Package (Biodex Medical System Inc., Shirley, NY, USA) | Single leg balance | Force platform (Advanced Mechanical Technologies Inc., Waltham MA, USA) |
| İnal et al. (2012) | DF/PF | A Cybex 6000 DYNAMOMETER | Linear sprint | New Test Photocell System 2000 (New Test OY) |
|  | IV/EV |  |  |  |
| Kadlubowski et al. (2024) | 1 RM Calf raise | Standing calf raise machine (Flame Sport,  Siauliai, Lithuania) | Linear sprint | Timing gates Brower TC Timing System, Biederitz, Germany |
|  |  |  | CMJ | Contact mat (Refitronic, Schmitten, Germany) |
|  |  |  | RSI |  |
| Kayhan et al. (2024) | PF/DF | Active Force Dynamometer (Version: 2, Digital Dynamometer, San Diego, United States) | Dynamic balance | Togu Challenge Disc (German Togu Company: Ger many) |
|  | IV/EV |  | RSI | Jump Mat Pro device (Bolzano, Bozen, Italy) |
| Kim and Park (2015) | Eccentric and Concentric DF | Biodex System  III (Biodex Medical Systems Inc., Shirley, USA) | Single leg stance | Biodex Balance System (Biodex Medical Systems Inc., Shirley, USA) |
|  | Eccentric and Concentric EV |  | Dynamic balance | Biodex Balance System (Biodex Medical Systems Inc., Shirley, USA) |
| Kouzaki and Shinohara (2010) | PF (MVC) | Strain gauge transducer (LTZ-200KA; Kyowa, Tokyo, Japan) | Bipedal quiet standing | Force platform (Type 9281B; Kistler, Zürich, Switzerland) |
|  |  |  |  | Surface electromyogram (SX203; Biometrics Ltd, Gwent, U.K.) |
| Kozinc et al. (2021) | PF/DF | Isometric dynamometers (S2P, Science to Practice, Ljubljana, Slovenia), embedded force sensors (model 1-Z6FC3/200kg or model PW2DC3/72KG, HBM, Darmstadt,  Germany) | COD | Single-beam photocell timing gates (Brower Timing Systems, Draper, UT, USA) |
| Möck et al. (2018) | 1 RM Calf raise | Two laser distance systems with a measuring range of 2000mmandameasuringrateof750Hz (model: optoNCDT1302–200, manufacture: Micro-Epsilon, Ortenburg, Germany. | Sprint test | Single-beam photocell timing gates (Brower Timing Systems, Draper, UT, USA) |
| Möck et al. (2023) | 1 RM Calf raise | Two laser distance systems with a measuring range of 2000mmandameasuringrateof750Hz (model: optoNCDT1302–200, manufacture: Micro-Epsilon, Ortenburg, Germany. | SJ | Contact mat measuring system (Refitronic, Schmit ten, Germany) |
|  |  |  | CMJ |  |
|  |  |  | RSI |  |
| Muehlbauer et al. (2013) | Isometric PF | Isokinetic system (Isomed 2000; D & R Ferstl GmbH, Hemau, Germany) | CoP | Balance platform (GKS 1000; IMM, Mittweida, Germany) |
|  |  |  | CMJ | Force platform (Kistler Type  9290AD; Kistler, Winterthur, Switzerland) |
| Muehlbauer et al. (2012) | Isometric PF | \  Isokinetic system (Isomed 2000; D & R Ferstl GmbH, Hemau, Germany) | Static balance | Balance platform (GKS 1000; IMM, Mittweida, Germany) |
|  |  |  | Dynamic Balance |  |
|  |  |  | CMJ | Force platform (Kistler Type  9290AD; Kistler, Winterthur, Switzerland) |
| Oshita and Yano (2012) | PF | Load cell (LPR-A-S10, Kyowa, Japan; 1 mV/V) | Postural Sway | Laser displacement sensor (ANR 1251, SUNX, Japan; 3.5 micro-m) |
| Ranisavljev et al. (2014) |  | Isokinetic dynamometer Kin-Com (Chattecx Corporation, Chattanooga, TN, USA) | Run to walk speed | Motorized treadmill (T200 Run Race, Technogym, Italy) |
|  | Isokinetic DF/PF |  |  |  |
| Sara et al. (2021) | Isometric PF | Biodex dynamometer (Biodex System 3 Pro; Biodex Medical; Shirley, NY | Single leg heel raise | Custom-made heels raise device |
| Shimizu et al. (2024) | PF | Isokinetic dynamometer (Cybex NORM, CSMi) | Rebound drop jump | Vertical-jump measurement scale (Yardstick, Swift Performance) |
|  |  |  | RDJ index |  |
| Singh et al. (2022) | PF/DF | Isokinetic dynamometer (Biodex Medical System Inc., Shirley, NY, USA) | Single leg hop jump | Kinematic Measurement System |
| Słomka and Michalska (2024) | PF/DF | Linear force transducer (SML Low Height S-type load cell, Interface®, Inc. Arizona, USA) | Forward functional stability index | NA |
|  |  |  | CoP displacement | Force plate (AMTI AccuGait, Watertown, MA, USA) |
| Genuario and Dolgener (1980) | PF | Isokinetic dynamometer (Cybex) | Vertical jump | NR |
| Tao et al. (2020) | IV/EV | Hand-held dynamometer (HHD; MicroFET 2, Hoggan  Scientific LLC, Salt Lake City, UT | Single leg balance | Modified Balance Error Scoring System |
| Trajković et al. (2021) | PF/DF | Isometric dynamometer (S2P, Science to Practice, Ljubljana, Slovenia [force sensor: model 1-Z6FC3/200 kg, HBM, Darmstadt, Germany] | Postural Stability | Piezoelectric force platform (model 9260AA, Kistler, Winterthur, Switzerland |
| Vecbērza et al. (2025) | PF | Isometric Unilateral PF | 20m sprint | (photocells; Muscle Lab, Ergo test Technology) |
|  |  |  | Unilateral RSI | Infrared optical contact grid (Muscle Lab, Ergo test Technology) |
| Wyrick (1969) | PF/DF | Cable tensiometer | Bass stick test | NA |
| Yoshizawa et al. (2020) | PF/DF | Handheld dynamometer (μTas F-1: Anima Co., Ltd., Tokyo, Japan) | Leg extension | Strength Ergo 240 (Mitsubishi Electric Engineering Company Limited) |
| Yoshizawa and Yoshida (2022) | PF | Handheld dynamometer (μTas F-1: Anima Co., Ltd., Tokyo, Japan) | Single leg stance | Body Pressure Measurement System (Nitta Corp., Osaka, Japan) |
| Chen et al. (2025) | Concentric PF | IsoMed-2000 isokinetic dynamometer | Horizontal deceleration | Radar device (Stalker ATS II, Applied Concepts, Inc., Dallas, TX, USA) |
| Note: PF- Plantar flexion, DF- Dorsiflexion, IV- Inversion, EV-Eversion, CMJ- Countermovement Jump, SJ- Squat Jump, RSI- Reactive Strength Index, COD- Change of Direction, RM- Repetition Maximum, NA- Not Applicable, NA- Not Reported | | | | |
