# Supplementary material for: Associations between ankle strength and physical performance in healthy individuals: a systematic review
Source: Front Physiol. 2026 Jul 15;17:1863201. doi: 10.3389/fphys.2026.1863201 (PMC13413676; doi:10.3389/fphys.2026.1863201)
Supplement: Supplementary file 3 [file SupplementaryFile3.docx]

|  | | | | |
| --- | --- | --- | --- | --- |
| **Supplementary file 3.** Significant and non-significant associations between the ankle strength and physical performance of the studies included in this review. | | | | |
|  | **Statistical Analysis** | **variables** | **value** | **p value** |
| Abd El-Kader (2014) | Pearson’s correlation | Static balance (berg balance test) with DF muscle force (control group) | 0.95 | NA |
|  |  | Flexibility (functional reach test) with DF muscle force (CG) | 0.96 | NA |
|  |  | Dynamic balance (timed to get up) with DF muscle force (CG) | -0.89 | NA |
|  |  | Static balance (berg balance test) with DF muscle force (training group) | 0.94 | NA |
|  |  | Flexibility (functional reach test) with DF muscle force (TG) | 0.95 | NA |
|  |  | Dynamic balance (timed to get up) with DF muscle force (TG) | -0.88 | NA |
| Cattagni et al. (2014) | Linear regression | PF/DF MIT with Normalized CoP (curvilinear) | 0.68 | 0.01 |
|  |  | PF/DF MIT with Normalized CoP (linear) | 0.6 | 0.01 |
|  |  | Non fallers PF/DF MIT with Normalized CoP (linear) | 0.4 | 0.01 |
|  |  | Elderly fallers PF/DF MIT with Normalized CoP (linear) | 0.55 | 0.01 |
| Chen et al. (2021) | Pearson’s correlation | PTq of PF with leg spring stiffness test | 0.561 | 0.01 |
|  |  | PTq of PF concentric with leg spring stiffness test | 0.571 | 0.012 |
|  |  | PTq of DF eccentric with leg spring stiffness test | 0.506 | 0.031 |
|  |  |  |  |  |
|  |  | PTq of PF eccentric with leg spring stiffness test | 0.436 | 0.014 |
| Cobb et al. (2014) | Multiple Regression Analysis | DF/PF, IV/EV with mediolateral postural stability | 0.098 | 0.05 |
|  |  | DF/PF, IV/EV with anteroposterior postural stability | 0.042 | 0.05 |
| Ding et al. (2024) |  | DF/PF, IV/EV with Anteroposterior postural stability | 0.042 | 0.05 |
|  |  | Ankle plantar flexor asymmetry and CMJ | −0.56 | 0.01 |
|  |  | PF Asymmetry with CMJ (boys) | -0.47 | 0.05 |
|  |  | PF Asymmetry with CMJ (girls) | -0.78 | 0.01 |
| Feehan et al. (2022) | Pearson’s correlation | **Left** |  |  |
|  |  | IV torque with Right Reaction time (RT) | -0.28 | 0.28 |
|  |  | EV torque with Right RT | -0.53 | 0.03 |
|  |  | IV torque with left RT | -0.26 | 0.31 |
|  |  | EV torque with left RT | -0.45 | 0.07 |
|  |  | **Right** |  |  |
|  |  | IV torque with Right Reaction time (RT) | -0.21 | 0.41 |
|  |  | EV torque with Right RT | -0.1 | 0.7 |
|  |  | IV torque with left RT | -0.24 | 0.02 |
|  |  | EV torque with left RT | 0.35 | 0.94 |
| Hagen et al. (2020) |  | **Old adults** |  |  |
|  | Pearson’s correlation | Evertor peak torque with AP-LOS (old adults) | 0.396 | 0.05 |
|  |  | Invertor peak torque with AP-LOS | 0.236 | 0.05 |
|  |  | Summed muscle strength with AP-LOS | 0.362 | 0.05 |
|  |  | Evertor peak torque with ML-LOS (old adults) | -0.027 | 0.05 |
|  |  | Invertor peak torque with ML-LOS | -0.152 | 0.05 |
|  |  | Summed muscle strength with ML-LOS | -0.109 | 0.05 |
|  |  | Evertor peak torque with CoG -Sway | 0 | 0.05 |
|  |  | Invertor peak torque with CoG -Sway | 0.097 | 0.05 |
|  |  | Summed peak torque with CoG -Sway | 0.174 | 0.05 |
|  |  | Evertor peak torque with CoG -velocity | -0.007 | 0.05 |
|  |  | Invertor peak torque with CoG -velocity | -0.195 | 0.05 |
|  |  | Summed peak torque with CoG -velocity | -0.124 | 0.05 |
|  |  | **Young adults** |  |  |
|  |  | Evertor peak torque with AP-LOS | 0.417* | 0.05 |
|  |  | Invertor peak torque with AP-LOS | 0.323 | 0.05 |
|  |  | Summed muscle strength with AP-LOS | 0.455* | 0.05 |
|  |  | Evertor peak torque with ML-LOS | 0.235 | 0.05 |
|  |  | Invertor peak torque with ML-LOS | 0.408* | 0.05 |
|  |  | Summed muscle strength with ML-LOS | 0.425* | 0.05 |
|  |  | Evertor peak torque with CoG -Sway | 0.063 | 0.05 |
|  |  | Invertor peak torque with CoG -Sway | 0.035 | 0.05 |
|  |  | Summed peak torque with CoG -Sway | 0.065 | 0.05 |
|  |  | Evertor peak torque with CoG -velocity | 0.067 | 0.05 |
|  |  | Invertor peak torque with CoG -velocity | 0.03 | 0.05 |
|  |  | Summed peak torque with CoG -velocity | 0.065 | 0.05 |
| Hébert-Losier et al. (2023) | Pearson’s correlation | CR Body weight power with 10m average sprint time | -0.531 | 0.034 |
|  |  | CR Body weight power with 10m best sprint time | -0.370 | 0.158 |
|  |  | CR weighted power with 10m average sprint time | -0.628 | 0.009 |
|  |  | CR weighted power with 10m best sprint time | -0.527 | 0.036 |
|  |  | Calf strength endurance repetitions with 10m average sprint time | -0.282 | 0.289 |
|  |  | Calf strength endurance repetitions with 10m best sprint time | -0.291 | 0.273 |
|  |  | Calf strength endurance total Displacement with 10m average sprint time | -0.505 | 0.046 |
|  |  | Calf strength endurance total Displacement repetitions with 10m best sprint time | -0.505 | 0.046 |
|  |  | Calf strength endurance total work with 10m average sprint time | -0.545 | 0.029 |
|  |  | Calf strength endurance total work repetitions with 10m best sprint time | 0.503 | 0.047 |
| Hirono et al. (2020) | Pearson’s correlation | **Stable platform** |  |  |
|  |  | COP with CV of force at 5% of MVT | 0.512 | 0.002 |
|  |  | COP with CV of force at 20% of MVT | 0.298 | 0.093 |
|  |  | COP with CV of force at 50% of MVT | -0.044 | 0.808 |
|  |  | COP with maximal strength | -0.298 | 0.093 |
|  |  | **unstable platform** |  |  |
|  |  | COP with CV of force at 5% of MVT | 0.276 | 0.121 |
|  |  | COP with CV of force at 20% of MVT | 0.458 | 0.007 |
|  |  | COP with CV of force at 50% of MVT | 0.331 | 0.06 |
|  |  | COP with maximal strength | 0.051 | 0.779 |
| Lin et al. (2009) | Pearson’s correlation | **Eyes open - dominant leg** |  |  |
|  |  | EV 30 °/s PTq/BW with average radial displacement | -0.1 | >0.05 |
|  |  | EV 30 °/s PTq/BW with 95% ellipse area | -0.11 | >0.05 |
|  |  | EV 30°/s PTq/BW with average velocity | -0.02 | >0.05 |
|  |  | IV 30°/s PTq/BW with average radial displacement | 0.05 | >0.05 |
|  |  | IV 30°/s PTq/BW with 95% ellipse area | 0.04 | >0.05 |
|  |  | IV 30°/s PTq/BW with average velocity | -0.12 | >0.05 |
|  |  | EV 120°/s PTq/BW with average radial displacement | -0.09 | >0.05 |
|  |  | EV 120°/s PTq/BW with 95% ellipse area | -0.03 | >0.05 |
|  |  | EV 120°/s PTq/BW with average velocity | 0.07 | >0.05 |
|  |  | IV 120°/s PTq/BW with average radial displacement | 0.13 | >0.05 |
|  |  | IV 120°/s PTq/BW with 95% ellipse area | 0.21 | >0.05 |
|  |  | IV 120°/s PTq/BW with average velocity | 0.04 | >0.05 |
|  |  | 30°/s IV/EV ratio of PTq/BW with average radial displacement | -0.15 | >0.05 |
|  |  | 30°/s IV/EV ratio of PTq/BW with 95% ellipse area | -0.16 | >0.05 |
|  |  | 30°/s IV/EV ratio of PTq/BW with average velocity | 0.12 | >0.05 |
|  |  | 120°/s IV/EV ratio of PTq/BW with average radial displacement | -0.25 | >0.05 |
|  |  | 120°/s IV/EV ratio of PTq/BW with 95% ellipse area | -0.32 | >0.05 |
|  |  | 120°/s IV/EV ratio of PTq/BW with average velocity | 0.01 | >0.05 |
|  |  | **Eyes open - non-dominant leg** |  |  |
|  |  | EV 30°/s PTq/BW with average radial displacement | -0.08 | >0.05 |
|  |  | EV 30°/s PTq/BW with 95% ellipse area | -0.11 | >0.05 |
|  |  | EV 30°/s PTq/BW with average velocity | -0.09 | >0.05 |
|  |  | IV 30°/s PTq/BW with average radial displacement | -0.01 | >0.05 |
|  |  | IV 30°/s PTq/BW with 95% ellipse area | 0.04 | >0.05 |
|  |  | IV 30°/s PTq/BW with average velocity | -0.04 | >0.05 |
|  |  | EV 120°/s PTq/BW with average radial displacement | 0.02 | >0.05 |
|  |  | EV 120°/s PTq/BW with 95% ellipse area | -0.03 | >0.05 |
|  |  | EV 120°/s PTq/BW with average velocity | 0.28 | >0.05 |
|  |  | IV 120°/s PTq/BW with average radial displacement | 0.02 | >0.05 |
|  |  | IV 120°/s PTq/BW with 95% ellipse area | 0.21 | >0.05 |
|  |  | IV 120°/s PTq/BW with average velocity | 0.27 | >0.05 |
|  |  | 30°/s IV/EV ratio of PTq/BW with average radial displacement | -0.08 | >0.05 |
|  |  | 30°/s IV/EV ratio of PTq/BW with 95% ellipse area | -0.16 | >0.05 |
|  |  | 30°/s IV/EV ratio of PTq/BW with average velocity | -0.01 | >0.05 |
|  |  | 120°/s IV/EV ratio of PTq/BW with average radial displacement | -0.27 | >0.05 |
|  |  | 120°/s IV/EV ratio of PTq/BW with 95% ellipse area | -0.32 | >0.05 |
|  |  | 120°/s IV/EV ratio of PTq/BW with average velocity | -0.17 | >0.05 |
|  |  | **Eye closed - dominant leg** |  |  |
|  |  | EV 30°/s PTq/BW with average radial displacement | 0.1 | >0.05 |
|  |  | EV 30°/s PTq/BW with 95% ellipse area | 0.15 | >0.05 |
|  |  | EV 30°/s PTq/BW with average velocity | 0.33 | >0.05 |
|  |  | IV 30°/s PTq/BW with average radial displacement | 0.18 | >0.05 |
|  |  | IV 30°/s PTq/BW with 95% ellipse area | 0.27 | >0.05 |
|  |  | IV 30°/s PTq/BW with average velocity | 0.33 | >0.05 |
|  |  | EV 120°/s PTq/BW with average radial displacement | 0.11 | >0.05 |
|  |  | EV 120°/s PTq/BW with 95% ellipse area | 0.3 | >0.05 |
|  |  | EV 120°/s PTq/BW with average velocity | 0.32 | >0.05 |
|  |  | IV 120°/s PTq/BW with average radial displacement | 0.08 | >0.05 |
|  |  | IV 120°/s PTq/BW with 95% ellipse area | 0.34 | >0.05 |
|  |  | IV 120°/s PTq/BW with average velocity | 0.17 | >0.05 |
|  |  | 30°/s IV/EV ratio of PTq/BW with average radial displacement | -0.08 | >0.05 |
|  |  | 30°/s IV/EV ratio of PTq/BW with 95% ellipse area | -0.21 | >0.05 |
|  |  | 30°/s IV/EV ratio of PTq/BW with average velocity | 0.03 | >0.05 |
|  |  | 120°/s IV/EV ratio of PTq/BW with average radial displacement | -0.01 | >0.05 |
|  |  | 120°/s IV/EV ratio of PTq/BW with 95% ellipse area | -0.24 | >0.05 |
|  |  | 120°/s IV/EV ratio of PTq/BW with average velocity | 0.01 | >0.05 |
|  |  | EYE CLOSED - Non-dominant leg |  |  |
|  |  | EV 30°/s PTq/BW with average radial displacement | 0.19 | >0.05 |
|  |  | EV 30°/s PTq/BW with 95% ellipse area | 0.15 | >0.05 |
|  |  | EV 30°/s PTq/BW with average velocity | 0.35 | >0.05 |
|  |  | IV 30°/s PTq/BW with average radial displacement | 0.3 | >0.05 |
|  |  | IV 30°/s PTq/BW with 95% ellipse area | 0.27 | >0.05 |
|  |  | IV 30°/s PTq/BW with average velocity | 0.35 | >0.05 |
|  |  | EV 120°/s PTq/BW with average radial displacement | 0.28 | >0.05 |
|  |  | EV 120°/s PTq/BW with 95% ellipse area | 0.3 | >0.05 |
|  |  | EV 120°/s PTq/BW with average velocity | 0.38 | >0.05 |
|  |  | IV 120°/s PTq/BW with average radial displacement | 0.28 | >0.05 |
|  |  | IV 120°/s PTq/BW with 95% ellipse area | 0.34 | >0.05 |
|  |  | IV 120°/s PTq/BW with average velocity | 0.28 | >0.05 |
|  |  | 30°/s IV/EV ratio of PTq/BW with average radial displacement | -0.17 | >0.05 |
|  |  | 30°/s IV/EV ratio of PTq/BW with 95% ellipse area | -0.21 | >0.05 |
|  |  | 30°/s IV/EV ratio of PTq/BW with average velocity | -0.06 | >0.05 |
|  |  | 120°/s IV/EV ratio of PTq/BW with average radial displacement | -0.13 | >0.05 |
|  |  | 120°/s IV/EV ratio of PTq/BW with 95% ellipse area | -0.24 | >0.05 |
|  |  | 120°/s IV/EV ratio of PTq/BW with average velocity | -0.07 | >0.05 |
| İnal et al. (2012) | Pearson’s correlation | ***male*** |  |  |
|  |  | 30°/s right DF average power with 100-m sprint time | -0.078 | >0.05 |
|  |  | 30°/s left DF average power with 100-m sprint time | -0.677 | >0.05 |
|  |  | 120°/s right DF average power with 100-m sprint time | -0.365 | >0.05 |
|  |  | 120°/s left DF average power with 100-m sprint time | -0.051 | >0.05 |
|  |  | 30°/s right PF average power with 100-m sprint time | -0.064 | >0.05 |
|  |  | 30°/s left PF average power with 100-m sprint time | -0.082 | >0.05 |
|  |  | 120°/s right PF average power with 100-m sprint time | 0.31 | >0.05 |
|  |  | 120°/s left PF average power with 100-m sprint time | -0.122 | >0.05 |
|  |  | 30°/s right IV average power with 100-m sprint time | -0.736 | >0.05 |
|  |  | 30°/s left IV average power with 100-m sprint time | 0.227 | >0.05 |
|  |  | 120°/s right IV average power with 100-m sprint time | -0.866 | 0.026 |
|  |  | 120°/s left IV average power with 100-m sprint time | -0.657 |  |
|  |  | 30°/s right EV average power with 100-m sprint time | -0.82 | 0.046 |
|  |  | 30°/s left EV average power with 100-m sprint time | -0.86 | 0.028 |
|  |  | 120°/s right EV average power with 100-m sprint time | -0.767 | >0.05 |
|  |  | 120°/s left EV average power with 100-m sprint time | -0.625 | >0.05 |
|  |  | ***Female*** |  |  |
|  |  | 30°/s right DF average power with 100-m sprint time | -0.583 | >0.05 |
|  |  | 30°/s left DF average power with 100-m sprint time | -0.677 | >0.05 |
|  |  | 120°/s right DF average power with 100-m sprint time | -0.975 | 0.005 |
|  |  | 120°/s left DF average power with 100-m sprint time | -0.836 | >0.05 |
|  |  | 30°/s right PF average power with 100-m sprint time | -0.144 | >0.05 |
|  |  | 30°/s left PF average power with 100-m sprint time | -0.274 | >0.05 |
|  |  | 120°/s right PF average power with 100-m sprint time | -0.627 | >0.05 |
|  |  | 120°/s left PF average power with 100-m sprint time | -0.122 | >0.05 |
|  |  | 30°/s right IV average power with 100-m sprint time | 0.509 | >0.05 |
|  |  | 30°/s left IV average power with 100-m sprint time | -0.507 | >0.05 |
|  |  | 120°/s right IV average power with 100-m sprint time | -0.042 | >0.05 |
|  |  | 120°/s left IV average power with 100-m sprint time | -0.675 | >0.05 |
|  |  | 30°/s right EV average power with 100-m sprint time | 0.027 | >0.05 |
|  |  | 30°/s left EV average power with 100-m sprint time | -0.469 | >0.05 |
|  |  | 120°/s right EV average power with 100-m sprint time | -0.156 | >0.05 |
|  |  | 120°/s left EV average power with 100-m sprint time | -0.171 | >0.05 |
| Kadlubowski et al. (2024) | Pearson’s correlation | 1RM CR with 5m linear sprint | -0.108 | >0.05 |
|  |  | 1RM CR with 10m linear sprint | -0.083 | >0.05 |
|  |  | 1RM CR with 30m linear sprint | -0.171 | >0.05 |
|  |  | 1RM CR with CMJ height | 0.057 | >0.05 |
|  |  | 1RM CR with RSI 15cm | 0.094 | >0.05 |
|  |  | 1RM CR with RSI 30cm | 0.162 | >0.05 |
|  |  | 1RM CR with RSI 45cm | 0.358 | <0.05 |
|  |  | 1RM CR with RSI 60cm | 0.456 | <0.05 |
|  |  | Relative 1RM CR with 5m linear sprint | 0.028 | >0.05 |
|  |  | Relative 1RM CR with 10m linear sprint | 0.018 | >0.05 |
|  |  | Relative 1RM CR with 30m linear sprint | -0.104 | >0.05 |
|  |  | Relative 1RM CR with CMJ height | -0.074 | >0.05 |
|  |  | Relative 1RM CR with RSI 15cm | 0.101 | >0.05 |
|  |  | Relative 1RM CR with RSI 30cm | 0.197 | >0.05 |
|  |  | Relative 1RM CR with RSI 45cm | 0.348 | <0.05 |
|  |  | Relative 1RM CR with RSI 60cm | 0.456 | <0.05 |
| Kayhan et al. (2024) | Pearson’s correlation | Dominant leg (DL) DF strength with DL balance | -0.382 | 0.002 |
|  |  | DL DF strength with non-dominant leg (NDL)balance | -0.107 | 0.518 |
|  |  | NDL DF strength with DL balance | -0.062 | 0.706 |
|  |  | NDL DF strength with NDL balance | -0.147 | 0.371 |
|  |  | DL DF strength with DL RSI | 0.326 | 0.043 |
|  |  | DL DF strength with NDL RSI | 0.239 | 0.322 |
|  |  | NDL DF strength with DL RSI | 0.598 | 0.05 |
|  |  | NDL DF strength with NDL RSI | 0.602 | 0.05 |
|  |  | DL PF strength with DL balance | -0.348 | 0.03 |
|  |  | DL PF strength with NDL balance | -0.062 | 0.709 |
|  |  | NDL PF strength with DL balance | -0.319 | 0.046 |
|  |  | NDL PF strength with NDL balance | -0.28 | 0.048 |
|  |  | DL PF strength with DL RSI | 0.26 | 0.11 |
|  |  | DL PF strength with NDL RSI | 0.309 | 0.047 |
|  |  | NDL PF strength with DL RSI | 0.446 | 0.004 |
|  |  | NDL PF strength with NDL RSI | 0.384 | 0.016 |
| Kim and Park (2015) | Pearson’s correlation | **Concentric Evertors** (PTq/BW) |  |  |
|  |  | DFE- Static balance with EV PTq at 30°/s | -0.587 | >0.05 |
|  |  | DFE-Dynamic balance with EV PTq at 30°/s | 0.096 | >0.05 |
|  |  | FW- Static balance with EV PTq at 30°/s | -0.387 | >0.05 |
|  |  | FW-Dynamic balance with EV PTq at 30°/s | -0.391 | >0.05 |
|  |  | MF- Static balance with EV PTq at 30°/s | -0.488 | >0.05 |
|  |  | MF-Dynamic balance with EV PTq at 30°/s | 0.125 | >0.05 |
|  |  | DFE- Static balance with EV PTq at 60°/s | -0.496 | >0.05 |
|  |  | DFE-Dynamic balance with EV PTq at 60°/s | -0.013 | >0.05 |
|  |  | FW- Static balance with EV PTq at 60°/s | -0.581 | >0.05 |
|  |  | FW-Dynamic balance with EV PTq at 60°/s | -0.592 | >0.05 |
|  |  | MF- Static balance with EV PTq at 60°/s | -0.264 | >0.05 |
|  |  | MF-Dynamic balance with EV PTq at 60°/s | 0.493 | >0.05 |
|  |  | DFE- Static balance with EV PTq at 90°/s | 0.377 | >0.05 |
|  |  | DFE-Dynamic balance with EV PTq at 90°/s | -0.082 | >0.05 |
|  |  | FW- Static balance with EV PTq at 90°/s | -0.522 | >0.05 |
|  |  | FW-Dynamic balance with EV PTq at 90°/s | 0.698 | >0.05 |
|  |  | MF- Static balance with EV PTq at 90°/s | -0.441 | >0.05 |
|  |  | MF-Dynamic balance with EV PTq at 90°/s | 0.413 | >0.05 |
|  |  | DFE- Static balance with EV PTq at 120°/s | -0.473 | >0.05 |
|  |  | DFE-Dynamic balance with EV PTq at 120°/s | 0.038 | >0.05 |
|  |  | FW- Static balance with EV PTq at 120°/s | -0.499 | >0.05 |
|  |  | FW-Dynamic balance with EV PTq at120°/s | -0.661 | >0.05 |
|  |  | MF- Static balance with EV PTq at 120°/s | -0.322 | >0.05 |
|  |  | MF-Dynamic balance with EV PTq at 120°/s | 0.384 | >0.05 |
|  |  | MF-Dynamic balance with EV PTq at 120°/s |  |  |
|  |  | **Eccentric Evertors** (PTq/BW) |  |  |
|  |  | DFE- Static balance with EV PTq at 30°/s | -0.621 | >0.05 |
|  |  | DFE-Dynamic balance with EV PTq at 30°/s | 0.504 | >0.05 |
|  |  | FW- Static balance with EV PTq at 30°/s | -0.275 | >0.05 |
|  |  | FW-Dynamic balance with EV PTq at 30°/s | -0.179 | >0.05 |
|  |  | MF- Static balance with EV PTq at 30°/s | -0.454 | >0.05 |
|  |  | MF-Dynamic balance with EV PTq at 30°/s | 0.166 | >0.05 |
|  |  | DFE- Static balance with EV PTq at 60°/s | -0.254 | >0.05 |
|  |  | DFE-Dynamic balance with EV PTq a 60°/s | 0.027 | >0.05 |
|  |  | FW- Static balance with EV PTq at 60°/s | -0.496 | >0.05 |
|  |  | FW-Dynamic balance with EV PTq at 60°/s | -0.563 | >0.05 |
|  |  | MF- Static balance with EV PTq at 60°/s | -0.338 | >0.05 |
|  |  | MF-Dynamic balance with EV PTq at 60°/s | 0.462 | >0.05 |
|  |  | DFE- Static balance with EV PTq at 90°/s | 0.039 | >0.05 |
|  |  | DFE-Dynamic balance with EV PTq at 90°/s | 0.012 | >0.05 |
|  |  | FW- Static balance with EV PTq at 90°/s | -0.818 | >0.05 |
|  |  | FW-Dynamic balance with EV PTq at 90°/s | -0.759 | >0.05 |
|  |  | MF- Static balance with EV PTq at 90°/s | -0.388 | >0.05 |
|  |  | MF-Dynamic balance with EV PTq at 90°/s | 0.353 | >0.05 |
|  |  | DFE- Static balance with EV PTq at 120°/s | -0.292 | >0.05 |
|  |  | DFE-Dynamic balance with EV PTq at 120°/s | 0.264 | >0.05 |
|  |  | FW- Static balance with EV PTq at 120°/s | -0.925 | <0.05 |
|  |  | FW-Dynamic balance with EV PTq at 120°/s | -0.91 | <0.05 |
|  |  | MF- Static balance with EV PTq at 120°/s | -0.253 | >0.05 |
|  |  | MF-Dynamic balance with EV PTq at 120°/s | 0.374 | >0.05 |
|  |  | **Eccentric Evertors** (WK/BW) |  |  |
|  |  | DFE- Static balance with EV PTq at 30°/s | 0.486 | >0.05 |
|  |  | DFE-Dynamic balance with EV PTq at 30°/s | 0.011 | >0.05 |
|  |  | FW- Static balance with EV PTq at 30°/s | -0.638 | >0.05 |
|  |  | FW-Dynamic balance with EV PTq at 30°/s | -0.645 | >0.05 |
|  |  | MF- Static balance with EV PTq at 30°/s | -0.335 | >0.05 |
|  |  | MF-Dynamic balance with EV PTq at 30°/s | 0.425 | >0.05 |
|  |  | DFE- Static balance with EV PTq at 60°/s | -0.609 | >0.05 |
|  |  | DFE-Dynamic balance with EV PTq at 60°/s | 0.044 | >0.05 |
|  |  | FW- Static balance with EV PTq at 60°/s | -0.667 | >0.05 |
|  |  | FW-Dynamic balance with EV PTq at 60°/s | -0.71 | >0.05 |
|  |  | MF- Static balance with EV PTq at 60°/s | -0.046 | >0.05 |
|  |  | MF-Dynamic balance with EV PTq at 60°/s | 0.425 | >0.05 |
|  |  | DFE- Static balance with EV PTq at 90°/s | -0.635 | >0.05 |
|  |  | DFE-Dynamic balance with EV PTq at 90°/s | 0.014 | >0.05 |
|  |  | FW- Static balance with EV PTq at 90°/s | -0.514 | >0.05 |
|  |  | FW-Dynamic balance with EV PTq at 90°/s | -0.698 | >0.05 |
|  |  | MF- Static balance with EV PTq at 90°/s | -0.29 | >0.05 |
|  |  | MF-Dynamic balance with EV PTq at 90°/s | 0.469 | >0.05 |
|  |  | DFE- Static balance with EV PTq at 120°/s | -0.552 | >0.05 |
|  |  | DFE-Dynamic balance with EV PTq at 120°/s | 0.108 | >0.05 |
|  |  | FW- Static balance with EV PTq at 120°/s | -0.502 | >0.05 |
|  |  | FW-Dynamic balance with EV PTq at 120°/s | -0.67 | >0.05 |
|  |  | MF- Static balance with EV PTq at 120°/s | -0.157 | >0.05 |
|  |  | MF-Dynamic balance with EV PTq at 120°/s | 0.475 | >0.05 |
|  |  | **Eccentric Evertors** (WK/BW) |  |  |
|  |  | DFE- Static balance with EV PTq at 30°/s | -0.628 | >0.05 |
|  |  | DFE-Dynamic balance with EV PTq at 30°/s | 0.636 | >0.05 |
|  |  | FW- Static balance with EV PTq at 30°/s | -0.542 | >0.05 |
|  |  | FW-Dynamic balance with EV PTq at 30°/s | -0.305 | >0.05 |
|  |  | MF- Static balance with EV PTq at 30°/s | -0.468 | >0.05 |
|  |  | MF-Dynamic balance with EV PTq at 30°/s | 0.136 | >0.05 |
|  |  | DFE- Static balance with EV PTq at 60°/s | -0.364 | >0.05 |
|  |  | DFE-Dynamic balance with EV PTq at 60°/s | 0.052 | >0.05 |
|  |  | FW- Static balance with EV PTq at 60°/s | -0.668 | >0.05 |
|  |  | FW-Dynamic balance with EV PTq at 60°/s | -0.708 | >0.05 |
|  |  | MF- Static balance with EV PTq at 60°/s | 0.638 | >0.05 |
|  |  | MF-Dynamic balance with EV PTq at 60°/s | -0.538 | >0.05 |
|  |  | DFE- Static balance with EV PTq at 90°/s | -0.071 | >0.05 |
|  |  | DFE-Dynamic balance with EV PTq at 90°/s | -0.006 | >0.05 |
|  |  | FW- Static balance with EV PTq at 90°/s | -0.852 | >0.05 |
|  |  | FW-Dynamic balance with EV PTq at 90°/s | -0.78 | >0.05 |
|  |  | MF- Static balance with EV PTq at 90°/s | -0.219 | >0.05 |
|  |  | MF-Dynamic balance with EV PTq at 90°/s | 0.307 | >0.05 |
|  |  | DFE- Static balance with EV PTq at 120°/s | -0.273 | >0.05 |
|  |  | DFE-Dynamic balance with EV PTq at 120°/s | 0.465 | >0.05 |
|  |  | FW- Static balance with EV PTq at 120°/s | -0.866 | >0.05 |
|  |  | FW-Dynamic balance with EV PTq at 120°/s | -0.857 | >0.05 |
|  |  | MF- Static balance with EV PTq at 120°/s | -0.139 | >0.05 |
|  |  | MF-Dynamic balance with EV PTq at 120°/s | 0.434 | >0.05 |
|  |  | **Concentric dorsiflexors** (PTq/BW) |  |  |
|  |  | DFE- Static balance with EV PTq at 30°/s | 0.221 | >0.05 |
|  |  | DFE-Dynamic balance with EV PTq at 30°/s | 0.348 | >0.05 |
|  |  | FW- Static balance with EV PTq at 30°/s | 0.216 | >0.05 |
|  |  | FW-Dynamic balance with EV PTq at 30°/s | 0.226 | >0.05 |
|  |  | MF- Static balance with EV PTq at 30°/s | 0.424 | >0.05 |
|  |  | MF-Dynamic balance with EV PTq at 30°/s | 0.12 | >0.05 |
|  |  | DFE- Static balance with EV PTq at 60°/s | -0.63 | >0.05 |
|  |  | DFE-Dynamic balance with EV PTq at 60°/s | 0.147 | >0.05 |
|  |  | FW- Static balance with EV PTq at 60°/s | 0.325 | >0.05 |
|  |  | FW-Dynamic balance with EV PTq at 60°/s | 0.363 | >0.05 |
|  |  | MF- Static balance with EV PTq at 60°/s | 0.897∗ | <0.05 |
|  |  | MF-Dynamic balance with EV PTq at 60°/s | -0.141 | >0.05 |
|  |  | DFE- Static balance with EV PTq at 90°/s | 0.125 | >0.05 |
|  |  | DFE-Dynamic balance with EV PTq at 90°/s | -0.603 | >0.05 |
|  |  | FW- Static balance with EV PTq at 90°/s | -0.637 | >0.05 |
|  |  | FW-Dynamic balance with EV PTq at 90°/s | -0.732 | >0.05 |
|  |  | MF- Static balance with EV PTq at 90°/s | 0.809 | >0.05 |
|  |  | MF-Dynamic balance with EV PTq at 90°/s | -0.473 | >0.05 |
|  |  | DFE- Static balance with EV PTq at 120°/s | 0.501 | >0.05 |
|  |  | DFE-Dynamic balance with EV PTq at 120°/s | 0.099 | >0.05 |
|  |  | FW- Static balance with EV PTq at120°/s | -0.068 | >0.05 |
|  |  | FW-Dynamic balance with EV PTq at 120°/s | 0.046 | >0.05 |
|  |  | MF- Static balance with EV PTq at 120°/s | 0.633 | >0.05 |
|  |  | MF-Dynamic balance with EV PTq at 120°/s | -0.676 | >0.05 |
|  |  | **Eccentric dorsiflexors** (PTq/BW) |  |  |
|  |  | DFE- Static balance with EV PTq at 30°/s | 0.223 | >0.05 |
|  |  | DFE-Dynamic balance with EV PTq at 30°/s | -0.366 | >0.05 |
|  |  | FW- Static balance with EV PTq at 30°/s | 0.043 | >0.05 |
|  |  | FW-Dynamic balance with EV PTq at 30°/s | 0.016 | >0.05 |
|  |  | MF- Static balance with EV PTq at 30°/s | 0.325 | >0.05 |
|  |  | MF-Dynamic balance with EV PTq at 30°/s | 0.001 | >0.05 |
|  |  | DFE- Static balance with EV PTq at 60°/s | -0.491 | >0.05 |
|  |  | DFE-Dynamic balance with EV PTq at 60°/s | 0.236 | >0.05 |
|  |  | FW- Static balance with EV PTq at 60°/s | -0.117 | >0.05 |
|  |  | FW-Dynamic balance with EV PTq at 60°/s | -0.062 | >0.05 |
|  |  | MF- Static balance with EV PTq at 60°/s | 0.575 | >0.05 |
|  |  | MF-Dynamic balance with EV PTq at 60°/s | -0.236 | >0.05 |
|  |  | DFE- Static balance with EV PTq at 90°/s | 0.008 | >0.05 |
|  |  | DFE-Dynamic balance with EV PTq at 90°/s | 0.199 | >0.05 |
|  |  | FW- Static balance with EV PTq at 90°/s | -0.892 | <0.05 |
|  |  | FW-Dynamic balance with EV PTq at 90°/s | 0.867 | >0.05 |
|  |  | MF- Static balance with EV PTq at 90°/s | 0.573 | >0.05 |
|  |  | MF-Dynamic balance with EV PTq at 90°/s | -0.087 | >0.05 |
|  |  | DFE- Static balance with EV PTq at 120°/s | 0.443 | >0.05 |
|  |  | DFE-Dynamic balance with EV PTq at 120°/s | 0.16 | >0.05 |
|  |  | FW- Static balance with EV PTq at 120°/s | -0.165 | >0.05 |
|  |  | FW-Dynamic balance with EV PTq at 120°/s | -0.137 | >0.05 |
|  |  | MF- Static balance with EV PTq at 120°/s | -0.334 | >0.05 |
|  |  | MF-Dynamic balance with EV PTq at 120°/s | -0.862 | <0.05 |
|  |  | **Concentric dorsiflexors** (WK/BW) |  |  |
|  |  | DFE- Static balance with EV PTq at 30°/s | 0.213 | >0.05 |
|  |  | DFE-Dynamic balance with EV PTq at 30°/s | -0.058 | >0.05 |
|  |  | FW- Static balance with EV PTq at 30°/s | 0.043 | >0.05 |
|  |  | FW-Dynamic balance with EV PTq at 30°/s | 0.114 | >0.05 |
|  |  | MF- Static balance with EV PTq at 30°/s | 0.246 | >0.05 |
|  |  | MF-Dynamic balance with EV PTq at 30°/s | 0.424 | >0.05 |
|  |  | DFE- Static balance with EV PTq at 60°/s | 0.562 | >0.05 |
|  |  | DFE-Dynamic balance with EV PTq at 60°/s | 0.347 | >0.05 |
|  |  | FW- Static balance with EV PTq at 60°/s | -0.173 | >0.05 |
|  |  | FW-Dynamic balance with EV PTq at 60°/s | 0.001 | >0.05 |
|  |  | MF- Static balance with EV PTq at 60°/s | 0.79 | >0.05 |
|  |  | MF-Dynamic balance with EV PTq at 60°/s | -0.294 | >0.05 |
|  |  | DFE- Static balance with EV PTq at 90°/s | -0.002 | >0.05 |
|  |  | DFE-Dynamic balance with EV PTq at 90°/s | -0.254 | >0.05 |
|  |  | FW- Static balance with EV PTq at 90°/s | 0.744 | >0.05 |
|  |  | FW-Dynamic balance with EV PTq at 90°/s | 0.767 | >0.05 |
|  |  | MF- Static balance with EV PTq at 90°/s | 0.827 | >0.05 |
|  |  | MF-Dynamic balance with EV PTq at 90°/s | -0.41 | >0.05 |
|  |  | DFE- Static balance with EV PTq at 120°/s | 0.456 | >0.05 |
|  |  | DFE-Dynamic balance with EV PTq at 120°/s | 0.192 | >0.05 |
|  |  | FW- Static balance with EV PTq at 120°/s | -0.466 | >0.05 |
|  |  | FW-Dynamic balance with EV PTq at 120°/s | -0.317 | >0.05 |
|  |  | MF- Static balance with EV PTq at 120°/s | 0.407 | >0.05 |
|  |  | MF-Dynamic balance with EV PTq at 120°/s | -0.807 | >0.05 |
|  |  | **Eccentric dorsiflexors** (WK/BW) |  |  |
|  |  | DFE- Static balance with EV PTq at 30°/s | 0.296 | >0.05 |
|  |  | DFE-Dynamic balance with EV PTq at 30°/s | -0.457 | >0.05 |
|  |  | FW- Static balance with EV PTq at 30°/s | 0.152 | >0.05 |
|  |  | FW-Dynamic balance with EV PTq at 30°/s | 0.132 | >0.05 |
|  |  | MF- Static balance with EV PTq at 30°/s | 0.546 | >0.05 |
|  |  | MF-Dynamic balance with EV PTq at 30°/s | 0.135 | >0.05 |
|  |  | DFE- Static balance with EV PTq at 60°/s | -0.448 | >0.05 |
|  |  | DFE-Dynamic balance with EV PTq at 60°/s | 0.325 | >0.05 |
|  |  | FW- Static balance with EV PTq at 60°/s | -0.226 | >0.05 |
|  |  | FW-Dynamic balance with EV PTq at 60°/s | –0.154 | >0.05 |
|  |  | MF- Static balance with EV PTq at 60°/s | 0.544 | >0.05 |
|  |  | MF-Dynamic balance with EV PTq at 60°/s | -0.066 | >0.05 |
|  |  | DFE- Static balance with EV PTq at 90°/s | 0.215 | >0.05 |
|  |  | DFE-Dynamic balance with EV PTq at 90°/s | -0.318 | >0.05 |
|  |  | FW- Static balance with EV PTq at 90°/s | -0.832 | >0.05 |
|  |  | FW-Dynamic balance with EV PTq at 90°/s | -0.773 | >0.05 |
|  |  | MF- Static balance with EV PTq at 90°/s | 0.715 | >0.05 |
|  |  | MF-Dynamic balance with EV PTq at 90°/s | -0.283 | >0.05 |
|  |  | DFE- Static balance with EV PTq at 120°/s | 0.475 | >0.05 |
|  |  | DFE-Dynamic balance with EV PTq at 120°/s | 0.147 | >0.05 |
|  |  | FW- Static balance with EV PTq at 120°/s | -0.374 | >0.05 |
|  |  | FW-Dynamic balance with EV PTq at 120°/s | -0.115 | >0.05 |
|  |  | MF- Static balance with EV PTq at 120°/s | -0.224 | >0.05 |
|  |  | MF-Dynamic balance with EV PTq at 120°/s | 0.919∗∗ | <0.01 |
| Kouzaki and Shinohara (2010) | Pearson’s correlation | cv of COP with PF EMG power at 10-15 Hz | 0.455 | <0.01 |
|  |  | cv of COP with CV of PF force at 2.5% of MVC | 0.62 | <0.001 |
|  |  | CV of COP with PF force at 5% of MVC | 0.455 | <0.001 |
| Kozinc et al. (2021) | Pearson’s correlation | **Basketball** |  |  |
|  |  | PF PTq with CoD 90 | -0.42 | <0.01 |
|  |  | PF RTD 50 with CoD 90 | -0.38 | <0.01 |
|  |  | PF RTD 10 0 with CoD 90 | -0.44 | <0.01 |
|  |  | PF PTq with CoD 180 | -0.38 | <0.01 |
|  |  | PF RTD 50 with CoD 180 | -0.37 | <0.01 |
|  |  | PF RTD 10 0 with CoD 180 | -0.38 | <0.01 |
|  |  | DF PTq with CoD 90 | -0.22 | <0.01 |
|  |  | DF RTD 50 with CoD 90 | -0.32 | <0.01 |
|  |  | DF RTD 10 0 with CoD 90 | -0.29 | <0.01 |
|  |  | DF PTq with CoD 180 | -0.29 | <0.01 |
|  |  | DF RTD 50 with CoD 180 | -0.41 | <0.01 |
|  |  | DF RTD 10 0 with CoD 180 | -0.39 | <0.01 |
|  |  | **Running** |  |  |
|  |  | PF PTq with CoD 90 | -0.3 | <0.05 |
|  |  | PF RTD 50 with CoD 90 | -0.19 | >0.05 |
|  |  | PF RTD 10 0 with CoD 90 | -0.3 | <0.05 |
|  |  | PF PTq with CoD 180 | -0.12 | >0.05 |
|  |  | PF RTD 50 with CoD 180 | -0.04 | >0.05 |
|  |  | PF RTD 10 0 with CoD 180 | -0.17 | >0.05 |
|  |  | DF PTq with CoD 90 | -0.22 | >0.05 |
|  |  | DF RTD 50 with CoD 90 | -0.05 | >0.05 |
|  |  | DF RTD 10 0 with CoD 90 | -0.09 | >0.05 |
|  |  | DF PTq with CoD 180 | -0.17 | >0.05 |
|  |  | DF RTD 50 with CoD 180 | -0.05 | >0.05 |
|  |  | DF RTD 10 0 with CoD 180 | -0.17 | >0.05 |
|  |  | **Tennis** |  |  |
|  |  | PF PTq with CoD 90 | -0.23 | <0.05 |
|  |  | PF RTD 50 with CoD 90 | -0.24 | <0.05 |
|  |  | PF RTD 10 0 with CoD 90 | -0.29 | <0.01 |
|  |  | PF PTq with CoD 180 | -0.39 | <0.01 |
|  |  | PF RTD 50 with CoD 180 | -0.36 | <0.01 |
|  |  | PF RTD 10 0 with CoD 180 | -0.37 | <0.01 |
|  |  | DF PTq with CoD 90 | -0.28 | <0.01 |
|  |  | DF RTD 50 with CoD 90 | -0.32 | <0.01 |
|  |  | DF RTD 10 0 with CoD 90 | -0.37 | <0.01 |
|  |  | DF PTq with CoD 180 | -0.48 | <0.01 |
|  |  | DF RTD 50 with CoD 180 | -0.39 | <0.01 |
|  |  | DF RTD 10 0 with CoD 180 | -0.47 | <0.01 |
| Möck et al. (2023) | Pearson’s correlation | Calf Raise 1 RM with SJ | 0.659 | <0.01 |
|  |  | CR 1 RM with CMJ | 0.708 | <0.01 |
|  |  | CR 1 RM with RSI 16 cm | 0.326 | <0.01 |
|  |  | CR 1 RM 24 cm | 379 | <0.01 |
|  |  | CR 1 RM 32 cm | 526 | <0.01 |
|  |  | CR 1 RM with 40 cm | 514 | <0.01 |
|  |  | CR 1 RM 48 cm | 457 | <0.01 |
|  |  | CR 1 RM relative with SJ | 575 | <0.01 |
|  |  | CR 1 RM relative with CMJ | 565 | <0.01 |
|  |  | CR 1 RM with relative RSI 16 cm | 436 | <0.01 |
|  |  | CR 1 RM relative with 24 cm | 472 | <0.01 |
|  |  | CR 1 RM relative with 32 cm | 573 | <0.01 |
|  |  | CR 1 RM relative with 40 cm | 535 | <0.01 |
|  |  | CR 1 RM relative with 48 cm | 521 | <0.01 |
| Möck et al. (2018) | Pearson’s correlation | CR 1 RM with 5m sprint time | -0.483 | <0.01 |
|  |  | CR 1 RM with 10m sprint time | -0.663 | <0.01 |
|  |  | CR 1 RM with 15m sprint time | -0.657 | <0.01 |
|  |  | CR 1 RM with 20m sprint time | -0.741 | <0.01 |
|  |  | CR 1 RM with 25m sprint time | -0.7 | <0.01 |
|  |  | CR 1 RM with 30m sprint time | -0.72 | <0.01 |
|  |  | CR 1 RM relative with 5m sprint time | -0.46 | <0.01 |
|  |  | CR 1 RM relative with 10m sprint time | -0.541 | <0.01 |
|  |  | CR 1 RM relative with 15m sprint time | -0.508 | <0.01 |
|  |  | CR 1 RM relative with 20m sprint time | -0.564 | <0.01 |
|  |  | CR 1 RM relative with 25m sprint time | -0.545 | <0.01 |
|  |  | CR 1 RM relative with 30m sprint time | -0.577 | <0.01 |
| Muehlbauer et al. (2013) | Pearson’s correlation | PF MIT with CoP AP(Static) | 0.178 | >0.05 |
|  |  | PF MIT with CoP ML S | 0.135 | >0.05 |
|  |  | PF MIT with CoP AP(Dynamic) | 0.126 | >0.05 |
|  |  | PF MIT with CoP ML D | 0.341 | >0.05 |
|  |  | PF RTD with CoP AP(Static) | 0.056 | >0.05 |
|  |  | PF RTD with CoP ML S | 0.041 | >0.05 |
|  |  | PF RTD with CoP AP(Dynamic) | 0.186 | >0.05 |
|  |  | PF RTD with CoP ML D | 0.387 | >0.05 |
|  |  | PF MIT with CMJ P | 0.458 | <0.05 |
|  |  | PF MIT with CMJ H | 0.511 | <0.01 |
|  |  | PF RTD with CMJ P | 0.689 | <0.01 |
|  |  | PF RTD with CMJ H | 0.628 | <0.01 |
| Muehlbauer et al. (2012) | Pearson’s correlation | PF MIT with CoP AP(Static) | 0.084 | >0.05 |
|  |  | PF MIT with CoP ML S | 0.105 | >0.05 |
|  |  | PF MIT with CoP AP(Dynamic) | 0.012 | >0.05 |
|  |  | PF MIT with CoP ML D | 0.173 | >0.05 |
|  |  | PF RTD with CoP AP(Static) | 0.201 | >0.05 |
|  |  | PF RTD with CoP ML S | 0.105 | >0.05 |
|  |  | PF RTD with CoP AP(Dynamic) | -0.033 | >0.05 |
|  |  | PF RTD with CoP ML D | 0.156 | >0.05 |
|  |  | PF MIT with CMJ P | 0.482 | <0.001 |
|  |  | PF MIT with CMJ H | 0.501 | <0.001 |
|  |  | PF RTD with CMJ P | 0.399 | <0.005 |
|  |  | PF RTD with CMJ H | 0.361 | <0.005 |
| Oshita and Yano (2012) | Pearson’s correlation | PF force fluctuation at 10% MVC with Postural sway | 0.58 | 0.05 |
|  |  | PF force fluctuation at 20% MVC with Postural sway | −.003 | 0.99 |
| Ranisavljev et al. (2014) | Pearson’s correlation | PF Isometric with WRT | 0.297 | > 0.05 |
|  |  | DF Isometric with WRT | 0.37 | <0.05 |
|  |  | PF Isokinetic60/s with WRT | 0.365 | <0.05 |
|  |  | DF Isokinetic60/s with WRT | 0.142 | > 0.05 |
|  |  | PF Isokinetic180/s with WRT | 0.157 | > 0.05 |
|  |  | DF Isokinetic180/s with WRT | -0.022 | > 0.05 |
|  |  | PF Isometric with RWT | 0.222 | > 0.05 |
|  |  | DF Isometric with RWT | 0.392 | <0.05 |
|  |  | PF Isokinetic60/s with RWT | 0.054 | > 0.05 |
|  |  | DF Isokinetic60/s with RWT | 0.022 | > 0.05 |
|  |  | PF Isokinetic180/s with RWT | 0.106 | > 0.05 |
|  |  | DF Isokinetic180/s with RWT | 0.029 | > 0.05 |
| Sara et al. (2021) | Pearson’s correlation | Baseline PF MVIC with SLHR reps | -0.005 | 0.979 |
|  |  | Post SLHR PF MVIC with SLHR reps | -0.234 | 0.23 |
| Shimizu et al. (2024) | Pearson’s correlation | ankle PF torque with CMJ height | 0.16 | > 0.05 |
|  |  | ankle PF torque with RDJ height | 0.31 | > 0.05 |
|  |  | ankle PF torque with RDJ Contact time (1st half) | -0.14 | > 0.05 |
|  |  | ankle PF torque with RDJ Contact time (2nd half) | 0.09 | > 0.05 |
|  |  | ankle PF torque with RDJ Contact time (total) | -0.01 | > 0.05 |
|  |  | ankle PF torque with RDJ index | 0.13 | > 0.05 |
| Singh et al. (2022) | Pearson’s correlation | DL PF (30°/s) with DL power | 0.83 | < 0.001 |
|  |  | DL DF (30°/s) with DL power | 0.56 | < 0.001 |
|  |  | DL PF (120°/s) with DL power | 0.85 | < 0.001 |
|  |  | DL DF (120°/s) with DL power | 0.73 | < 0.001 |
|  |  | NDL PF (30°/s) with NDL power | 0.61 | 0.001 |
|  |  | NDL PF (30°/s) with NDL power | 0.41 | 0.2 |
|  |  | NDL PF (120°/s) with NDL power | 0.56 | 0.002 |
|  |  | NDL PF (120°/s) with NDL power | 0.32 | 0.6 |
| Słomka and Michalska (2024) | Pearson’s correlation | COP D with left DF | 0.34 | > 0.05 |
|  |  | COP D with left PF | 0.5 | < 0.05 |
|  |  | COP D with right DF | 0.41 | < 0.05 |
|  |  | COP D with right PF | 0.45 | < 0.05 |
|  |  | FFSI with left DF | 0.08 | > 0.05 |
|  |  | FFSI with left PF | 0.32 | > 0.05 |
|  |  | FFSI with right DF | 0.29 | > 0.05 |
|  |  | FFSI with right PF | 0.3 | > 0.05 |
| Genuario and Dolgener (1980) | Pearson’s correlation | VJ with PF (slow PTq) | 0.173 | > 0.05 |
|  |  | VJ with PF (fast PTq) | 0.238 | > 0.05 |
|  |  | RVJ with PF (slow PTq) | 0.424 | < 0.05 |
|  |  | RVJ with PF (fast PTq) | 0.502 | < 0.01 |
| Tao et al. (2020) | Pearson’s correlation | SLB stable and IV | -0.39 | > 0.05 |
|  |  | SLB stable and EV | -0.13 | > 0.05 |
|  |  | SLB unstable and IV | -0.08 | > 0.05 |
|  |  | SLB unstable and EV | -0.19 | > 0.05 |
|  |  | mBESS composite and IV | -0.07 | > 0.05 |
|  |  | mBESS composite and EV | -0.19 | > 0.05 |
| Trajković et al. (2021) | Pearson’s correlation | PF and CoP Total | 0.14 | < 0.01 |
|  |  | DF and CoP Total | 0.07 | < 0.05 |
|  |  | PF and CoP velocity anterior posterior | 0.17 | < 0.01 |
|  |  | DF and CoP velocity anterior posterior | 0.06 | > 0.05 |
|  |  | PF and CoP velocity medial lateral | 0.1 | < 0.01 |
|  |  | DF and CoP velocity medial lateral | 0.08 | < 0.05 |
|  |  | PF and CoP amplitude AP | 0.16 | < 0.01 |
|  |  | DF and CoP amplitude AP | 0.04 | > 0.05 |
|  |  | PF and COP amplitude ML | 0.07 | < 0.05 |
|  |  | PF and CoP amplitude ML | 0.01 | > 0.05 |
|  |  | PF and CoP frequency AP | 0.16 | < 0.01 |
|  |  | DF and CoP frequency AP | 0.04 | > 0.05 |
|  |  | PF and COP frequency ML | 0.07 | < 0.05 |
|  |  | PF and CoP frequency ML | 0.01 | > 0.05 |
| Vecbērza et al. (2025) | Pearson’s correlation | relative PF with 20m linear sprint | -0.36 | <0.05 |
|  |  | relative PF with unilateral RSI | 0.35 | <0.05 |
| Wyrick (1969) | Pearson’s correlation | PF and bass stick balance | -0.2 | <0.01 |
|  |  | PF and bass stick balance without visual cues | -0.23 | <0.01 |
|  |  | PF and high bass stick balance | -0.14 | <0.01 |
|  |  | DF and bass stick balance | -0.11 | <0.01 |
|  |  | DF and bass stick balance without visual cues | -0.09 | <0.01 |
|  |  | DF and high bass stick balance | -0.09 | <0.01 |
| Yoshizawa et al. (2020) | Pearson’s correlation | Right leg PF strength with right Leg extensor torque | 0.41 | <0.05 |
|  |  | Left leg PF strength with left Leg extensor torque | 0.45 | <0.05 |
|  |  | Right leg PF strength with right Leg extensor torque | 0.8 | <0.01 |
|  |  | Left leg PF strength with left Leg extensor torque | 0.65 | <0.01 |
| Yoshizawa and Yoshida (2022) | Pearson’s correlation | Total trajectory length (cm) and PF | −0.41 | <0.05 |
|  |  | Outer peripheral area (cm2) and PF | −0.55 | <0.05 |
| Chen et al. (2025) | Pearson’s correlation | Concentric PF PTq NDL with 5m Average deceleration | −0.52 | <0.001 |
|  |  | Concentric PF PTq DL with 5m Average deceleration | −0.53 | <0.001 |
|  |  | Concentric PF PTq 10 m Sprint time | -0.497 | 0.039 |
|  |  | Concentric PF PTq 30 m Sprint time | -0.578 | 0.013 |
|  |  | Concentric DF PTq with 10m sprint time | -0.461 | 0.059 |
|  |  | Concentric DF PTq with 30m sprint time | -0.461 | 0.059 |
|  |  | Eccentric DF PTq with 10m sprint time | -0.399 | 0.107 |
|  |  | Eccentric DF PTq with 30m sprint time | -0.438 | 0.075 |
| **Note**: NR- Not reported, DF-Dorsiflexion, PF-Plantarflexion, IV- Inversion, EV- Eversion, MIT- Maximal isometric torque, CoP- Center of pressure, PTq- Peak torque, CMJ- Countermovement jump, SJ- Squat Jump, nMIT- normalized maximal isometric torque, MVIC- maximal voluntary isometric contraction, LOS- limits of stability, AP- Anteroposterior, ML- Mediolateral, CoG- Center of gravity, OA- Older adults, BW- Body weight, MVT- maximal voluntary contraction, D- Dominant, ND-Non-dominant, SLB- Single leg balance, OE- Open eyes, CE-closed eyes, RM- Repetition maximum, RSI- Reactive strength index, FW- Forward, MF- Midfielder, RTD- Rate of torque development, CR-Calf raise, WRT- Walk to run, RWT- Run to walk, RDJ- Rebound drop jump, M-Male, F-Female, mBESS- Modified balance error scoring system, VSS-Visual squat score, FFSI- Forward functional stability index | | | | |
