# Supplementary material for: Associations between ankle strength and physical performance in healthy individuals: a systematic review
Source: Front Physiol. 2026 Jul 15;17:1863201. doi: 10.3389/fphys.2026.1863201 (PMC13413676; doi:10.3389/fphys.2026.1863201)
Supplement: Supplementary file 4 [file SupplementaryFile4.docx]

| **Supplementary file 4.** Search query used in the specific databases. | |
| --- | --- |
| **Database** | **Query** |
| **Scopus** | ( TITLE-ABS-KEY ( ankle AND strength ) OR TITLE-ABS-KEY ( dorsiflexor AND strength ) OR TITLE-ABS-KEY ( plantarflexor AND strength ) OR TITLE-ABS-KEY ( invertor AND strength ) OR TITLE-ABS-KEY ( evertor AND strength ) OR TITLE-ABS-KEY ( foot AND strength ) OR TITLE-ABS-KEY ( foot AND muscle ) OR TITLE-ABS-KEY ( ankle AND muscles ) AND TITLE-ABS-KEY ( performance ) OR TITLE-ABS-KEY ( physical AND fitness ) OR TITLE-ABS-KEY ( sport AND performance ) ) |
| **Web of Science** | (ankle strength (Topic) or dorsiflexor strength (Topic) or plantarflexor strength (Topic) or invertor strength (Topic) or evertor strength (Topic) and performance (Topic)) |
| **PubMed** | ((((((ankle strength) OR (dorsiflexor strength)) OR (plantar flexor strength)) OR (invertor strength)) OR (evertor strength)) AND (performance)) |
|  | ((((((ankle strength) OR (dorsiflexor strength)) OR (plantar flexor strength)) OR (invertor strength)) OR (evertor strength)) AND (Physical fitness)) |
